# Indacaterol inhibits collective cell migration and IGDQ-mediated single cell migration in metastatic breast cancer MDA-MB-231 cells

**DOI:** 10.1186/s12964-023-01340-9

**Published:** 2023-10-30

**Authors:** Sophie Ayama-Canden, Rodolfo Tondo, Martha Liliana Pineros Leyton, Noëlle Ninane, Catherine Demazy, Marc Dieu, Antoine Fattaccioli, Aude Sauvage, Tijani Tabarrant, Stéphane Lucas, Davide Bonifazi, Carine Michiels

**Affiliations:** 1 URBC – NARILIS, University of Namur, Rue de Bruxelles 61, 5000 Namur, Belgium; 2 Cardiff University, Park Place, Main Building, Wales, CF10 3AT UK; 3 MaSUN, Mass Spectrometry Facility, University of Namur, 61, Rue de Bruxelles, 5000 Namur, Belgium; 4 LARN – NARILIS, University of Namur, Rue de Bruxelles 61, Namur, 5000 Belgium; 5 Institute of Organic Chemistry, University of Vienna, Währinger Str. 38, 1090 Vienna, Austria

**Keywords:** SRFS6, Indacaterol, Breast cancer, Cell migration, Metastasis, IGDQ motogenic motif, Fibronectin type I, Integrin alpha 5, Integrin beta 3, Motogenic

## Abstract

Metastasis is the main cause of deaths related to breast cancer. This is particular the case for triple negative breast cancer. No targeted therapies are reported as efficient until now. The extracellular matrix, in particular the fibronectin type I motif IGDQ, plays a major role in regulating cell migration prior metastasis formation. This motif interacts with specific integrins inducing their activation and the migratory signal transduction.Here, we characterized the migratory phenotype of MDA-MB-231 cells, using functionalized IGDQ-exposing surfaces, and compared it to integrin A5 and integrin B3 knock-down cells. A multiomic analysis was developed that highlighted the splicing factor SRSF6 as a putative master regulator of cell migration and of integrin intracellular trafficking. Indacaterol-induced inhibition of SRSF6 provoked: i) the inhibition of collective and IGDQ-mediated cell migration and ii) ITGA5 sequestration into endosomes and lysosomes. Upon further studies, indacaterol may be a potential therapy to prevent cell migration and reduce metastasis formation in breast cancer.

Video Abstract

**Supplementary Information:**

The online version contains supplementary material available at 10.1186/s12964-023-01340-9.

## Introduction

Breast cancer is the most frequent type of cancer in women, with 24% in term of incidence. Breast cancers are distinguished in multiple subtypes according to their invasiveness but also to molecular and histological characteristics. Most of breast cancers that metastasize are triple negative breast cancer (TNBC) [1]. TNBC is the most aggressive type breast cancer with a poor prognosis and high metastatic potential. It is so named because of the absence of expression of three proteins: Human Epidermal Proliferative Receptor 2 (HER2), Estrogen Receptors (ER) and Progesterone Receptors (PR). Currently no specific therapy exists; thus conventional chemotherapy is used. Understanding the mechanisms behind metastatization is thus necessary to improve patient care and to develop new diagnostic tools and treatments.

Cancer metastasis begins with the detachment of metastatic cells from the primary tumor. Then cells travel to different sites through blood/lymphatic vessels, settle and grow at a distal site [2]. During this process, metastatic cells undergo detachment from the tumor, start migrating, must evade from the immune system, have to survive the anoikis and the hostile conditions within the blood flow and adapt to the new environment of the premetastatic niche [3]. Among these different steps, cell migration is one of the most important. Cell migration is regulated by tumor microenvironment components, including the composition of the extracellular matrix (ECM) [4]. Fibronectin (Fn), one of the major components of the ECM, is described to sustain cell migration via its interactions with transmembrane integrins. Fn is ubiquitously present in all tissues but is mostly secreted by the stromal fibroblasts. From a single gene, multiple variants are produced due to alternative splicing. These variants contain modules and motifs that are fulfilling different functions. Three types of modules have been described: type I, type II and type III [5]. Each module activates specific integrins inducing specific cell responses. Fibronectin deregulation in quantity and/or in type which is produced has been related to tumor progression and angiogenesis [6–10].

Specific Fn motifs shown to support breast cancer cell interact with integrins α5β1 and αvβ3. These two integrins are described as essential for cell migration and invasion [2, 11, 12]. These integrins can be extrinsically activated by the Fn type III proliferative RGD motif, Fn type I motogenic IGD motif or intrinsically by the activation of the EGFR pathway. However, the exact mechanism underlying the modulation of cell migration upon Fn to integrin binding is not yet clear.

**Fig. 1:**
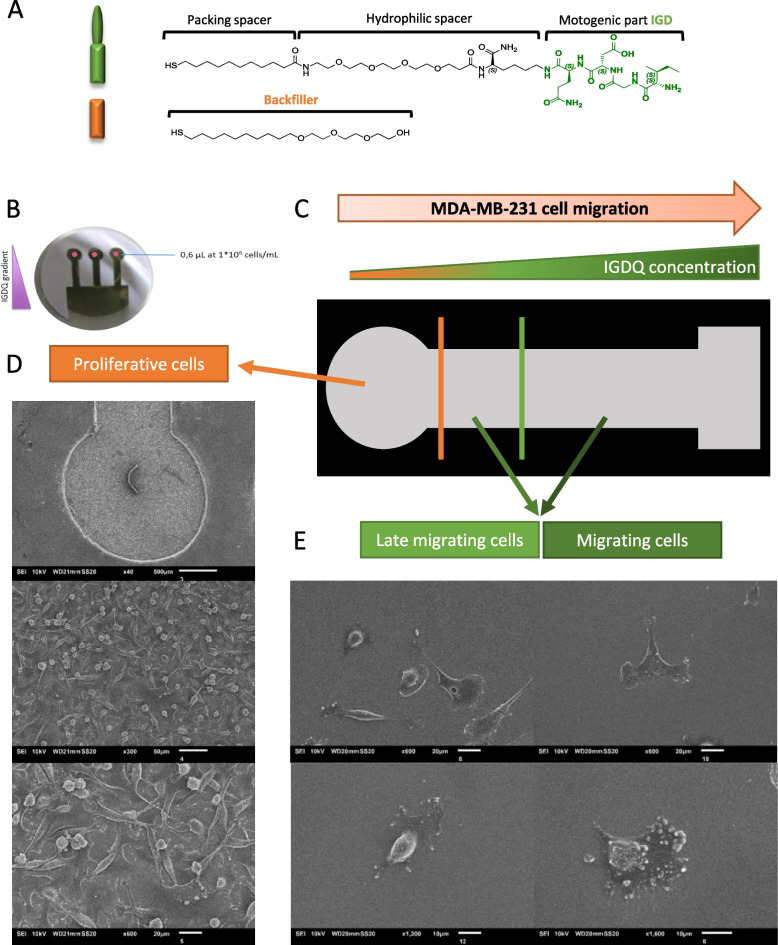
IGDQ-mediated cell migration along engineered surfaces. **A** Chemical structure of the peptides used for the gradient with the fibronectin motif IGDQ and the tetraethylenglycol as backfiller; **B** IGDQ-exposing surface pictures; **C** Schematic representation of engineered surface after 5 days of cell migration; Scanning electron microscopy images of MDA-MB-231 cells after 5 days of migration along engineered surface with **D** proliferative phenotype and **E** migrating phenotype

Using peptide-assisted cellular migration along engineered surfaces, we have recently shown in an in vitro model of cell migration that IGDQ-exposing type I fibronectin motif monolayers (SAMs) sustain the adhesion of MDA-MB-231 cells, which are a TNB cell line [4, 13]. The biochemical pathways mediating cell activation by IGD motif are still unclear and not well understood, further studies revealed that longer IGD-containing peptides present higher motogenic activity than peptides including only the smallest active unit IGD [14]. It was further demonstrated that IGD peptides stimulated fibroblast migration in the following order of activity IGDS > IGDQ > IGD through an initial cell activation process followed by a subsequent period of enhanced migration [14]. The biological responses interfaced with the SAM gradients show that only those exposing the IGDQ sequence induced significant migration of MDA-MB-231 cells. In particular, the observed migratory behavior suggests the presence of cell subpopulations associated with a “proliferating”, a “late migratory” or a “migratory” phenotype, the latter determining a considerable cell migration at the sub-cm length scale (Fig. 1).

In this work, IGDQ-exposing surfaces were used to separate MDA-MB-231 cells regarding their migrating phenotype (Fig. 1). The subpopulations either with a “proliferating” phenotype or with a “migratory” phenotype obtained with these engineered surfaces were characterized by a deep RNA-sequencing in order to identify new pathways regulating the metastasis process. To identify pathways and genes involved in both migration and integrin signaling, a multiomic approach was developed with the RNAseq data coupled with previous proteomic data obtained on ITGA5 or ITGB3 shRNA invalidated cells. A gene ontology (GO) analysis comparison allowed to highlight cell signaling pathways related to ITGA5, ITGB3 and IGDQ-mediated single cell migration. An in silico validation was performed for some top genes identified by this complex comparison identifying Serine And Arginine Rich Splicing Factor 6 (SRSF6), as a key actor of cell migration [15]. Moreover, since a recent study identified indacaterol, a chronic obstructive pulmonary disease (COPD) treatment, as an SRSF6 protein inhibitor [15], we targeted this protein with this molecule and studied the effect of its inhibition on cell migration.

## Results

### Integrin / IGDQ-mediated migratory phenotype determination

**Fig. 2:**
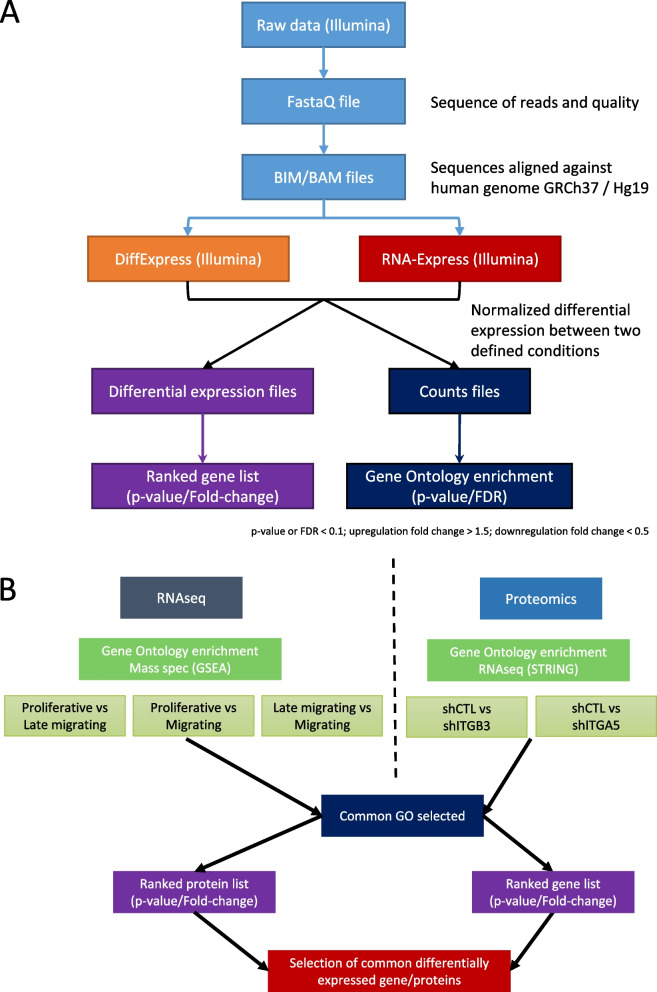
Multi-omic analysis protocol. **A** RNA sequencing raw data bioinformatics analysis management; **B** RNAseq and proteomic multi-omics data management

To determine the migratory phenotype on IGDQ-exposing surfaces (Fig. 1 – A), MDA-MB-231 cells were seeded on engineered surfaces at day one at the lowest IGDQ peptide concentration spot and incubated for 5 days (Fig. 1 – B). Three phenotypes were obtained: “proliferative” for proliferating cells at low IGDQ concentration (Fig. 1 – C), “late migratory” for cells that started late to follow the peptide gradient and “migratory” for migratory cells that early followed the IGDQ gradient concentration (Fig. 1 – D). At this step, cells were collected separately according to their phenotype. Because the number of “late migratory” and “migratory” cells obtained were low, cells from 16 engineered surfaces were pooled to obtain a range of 100 – 1000 cells for each sample. For each condition, four independent biological replicates were generated. Each replicate was then used to produce the library needed to perform the low-input RNA sequencing and raw data were analyzed as presented Fig. 2 – A.

**Fig. 3:**
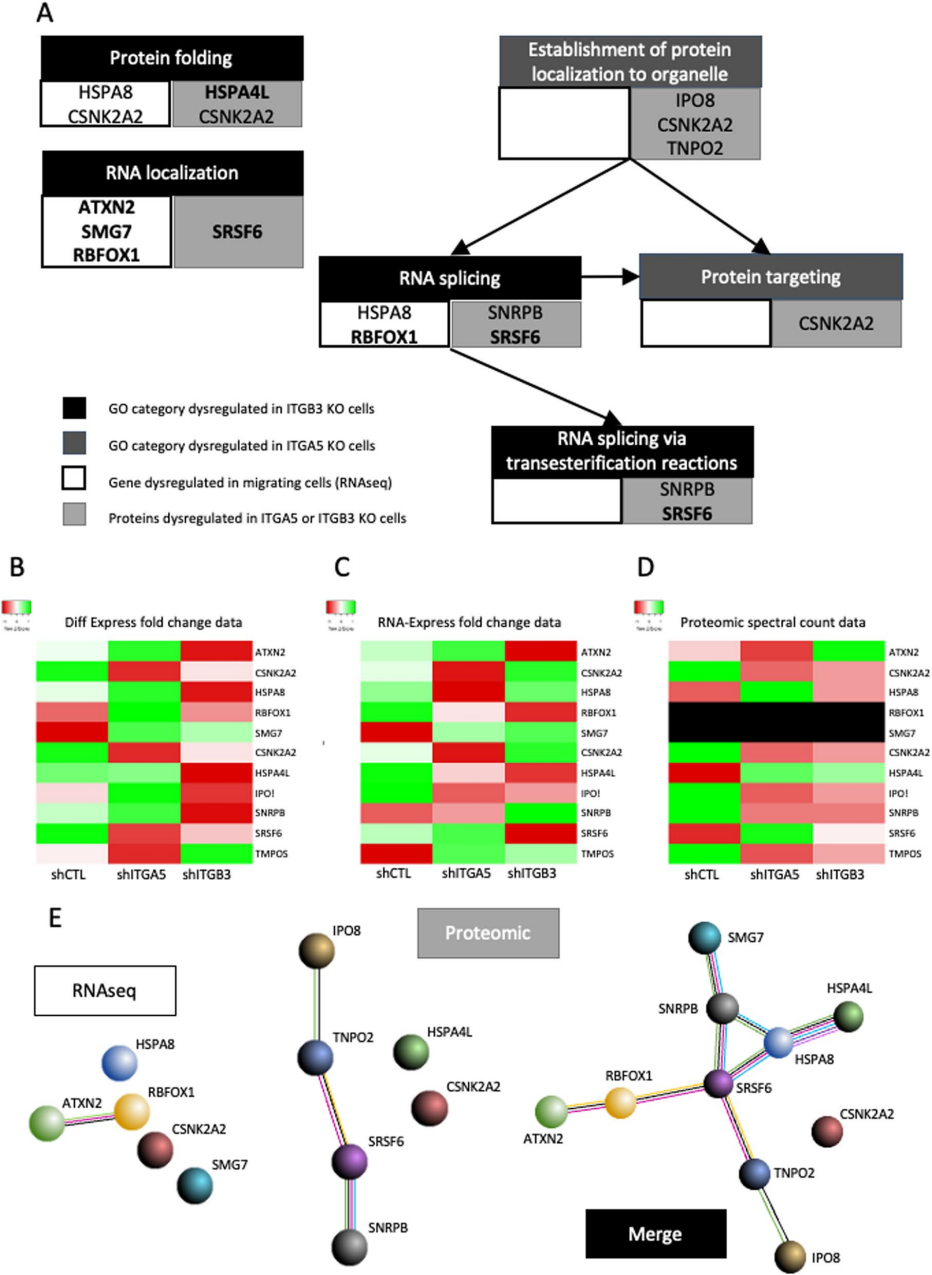
Multi-omics relative differential expression of the 10 selected targets. **A** Common gene ontology categories highlighted in both migrating cells and in ITGA5 or ITGB3 invalidated cells (*Cytoscape-GoClue* – used on 2020–08); **B** RNA differential expression of the 10 targets obtained using DiffEpress tool (Illumina); **C** RNA differential expression of the 10 targets obtained using RNA-Express tool (Illumina); **D** Protein level expression of the 10 targets obtained in shCTL, shITGA5 and shITGB3 cells; **E** String map of relevant genes and proteins obtained from RNAseq and proteomic analysis (*STRING* Expasy tool – used on 2020–08)

A meta-analysis was performed comparing this RNA-seq GO results to integrin α5 (ITGA5) and integrin β3 (ITGB3) related proteomic GO results obtained previously in our lab (Fig. 2 – B) [13]. The gene sets of common GO categories were compared to differentially expressed RNAs and proteins found in RNA-seq and proteomic analysis for all conditions (Fig. 3 – A). This step permitted to determine the GO categories and genes impacted both in cell migration and related to ITGA5 or ITGB3 knock-down. Two major biological processes were found impacted: “RNA splicing” and “protein folding and localization”.

From these two GO categories, 5 genes and 6 proteins were identified, with significative modified expression in migrating cells for the RNA seq and in ITGA5 or ITGB3 invalidated cells for the proteomic (Fig. 3 – Additional table 1). This differential expression is presented in heatmaps using data from RNA Express tool (Fig. 3 – B) and Differential expression tool (Fig. 3 – C) with A. proliferating cells vs late migratory, B. proliferating cells vs migratory cells and C. late migratory vs migratory cells. Counts from proteomic data, obtained during our previous study, are presented for control cells (shCTL), integrin alpha 5 invalidated cells (shITGA5) and integrin beta 3 invalidated cells (shITGB3) (Fig. 3 – D). Interactions between.

Except for one, all targets can be directly linked, meaning that the same major signalling pathways are involved in both migration and ITGA5 or ITGB3 related phenotypes. The quantitative data for RNAseq and proteomic data are presented in Additional table 1. The description of the highlighted genes in Fig. 3 are presented in Additional table 2.

Serine/arginine-rich splicing factor 6 (SRSF6) is part of one of the major class of splicing factors and involved in constitutive and alternative splicing of mRNA [16]. SRSF6 was found upregulated in both shITAG5 (FC = -0.77) and shITGB3 (FC = -0.38) invalidated cells. SRSF6 upregulation was related to promotion of cell proliferation and cell migration in MCF-10A breast cancer cells [17]. Recent study also related SRSF6 expression level in peripheral blood mononuclear cells (PBMCs) as a potential biomarker of the presence of metastases in breast cancer patients [18]. In colorectal cancer (CRC), SRSF6 was described to have an important role in alternative splicing mediating cancer progression. SRSF6 is considered as an unfavorable prognostic marker in renal and liver cancer [19]. Curently, targeting SRSF6 in cancer is studies using multiple strategies, such as small molecules inhibitors, siRNAs, decoy RNAs or antisense oligonucleotides [20]. A new antitumor drug targeting SRSF6 was found in CRC: indacaterol, an adrenergic receptor 2 agonist (ADRB2) [15].

### Inhibition of cell migration by indacaterol SRSF6 blockade

After a deep literature analysis for each candidate, the splicing factor SRSF6 was highlighted as a good potential therapeutic target with regards to its role in proliferation and invasion in breast cancer [17], as unfavourable prognostic marker in multiple cancer types [19] and due to the existence of molecules to target it. Moreover, SRSF6 was found to be upregulated in breast cancer cell lines and human tumors [17]. Wan & al. identified indacaterol, from an in silico screening in a panel of 4855 FDA-approved drugs using homology modelling (Drugbank database), as a direct inhibitor of SRSF6 protein. It acts by blocking SRSF6 second RNA-recognition motif (RRM2) [15]. Indacaterol is a β2-adrenergic (ADRB2) receptor agonist, commonly used to treat chronic obstructive pulmonary disease (COPD). The appropriate concentration of 15 µM of indacaterol was determined in our cell model (Additional file 1), in order not to induce cell death.

**Fig. 4:**
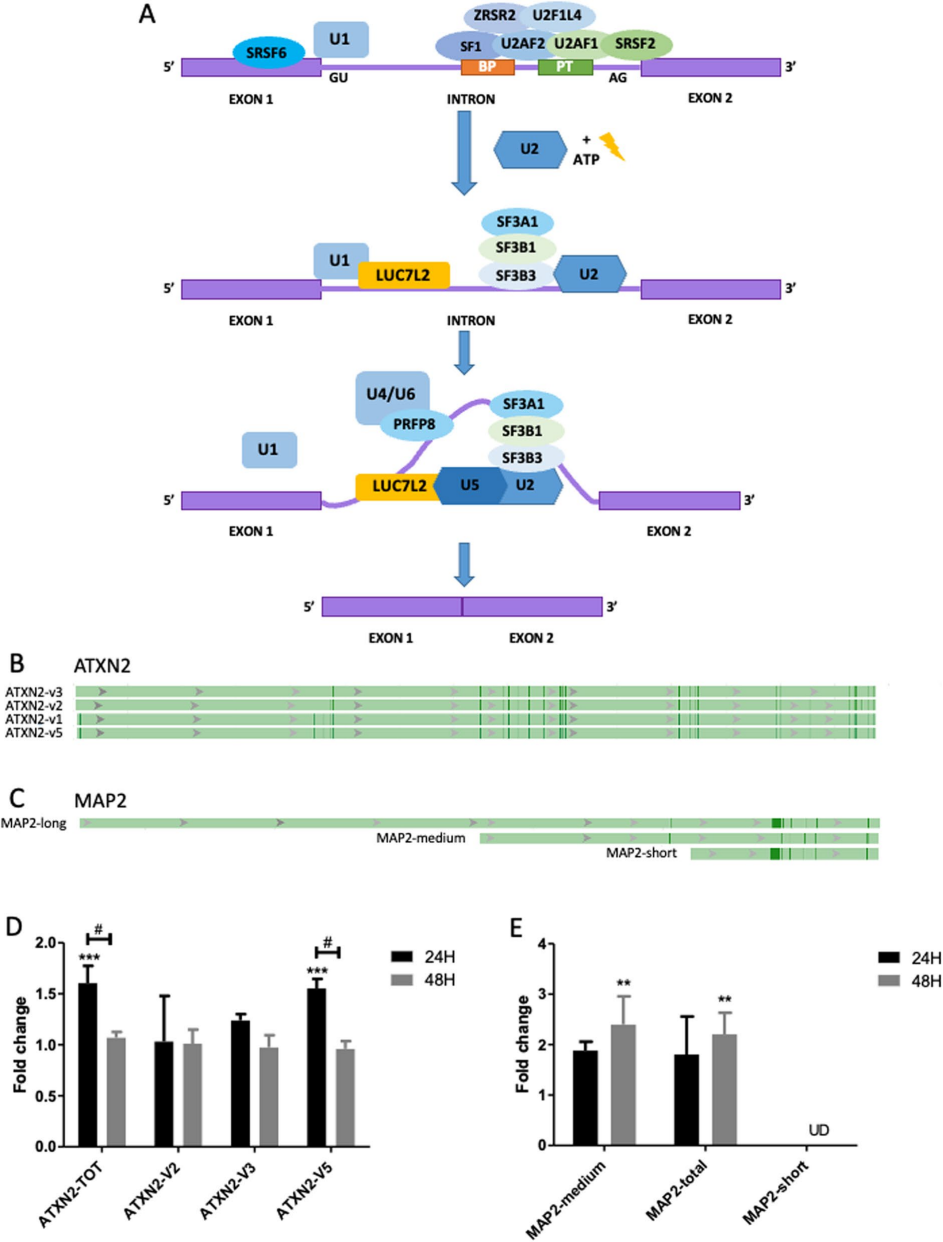
Validation of the inhibition of SRSF6 by Indacaterol in MDA-MB-231 cells. **A** Spliceosome complex SRSF6 dependent (adapted from Visconte *and al* [21]*.*). U1 snRNP: splicing recruitment initiation protein; SRSF6: 5′splicing site serine-arginine rich (SR) protein regulating alternative splicing; SF1 U2 auxiliary factor subunit 2 U2AF2: bind the branch point (BP) sequence and the polypyrimidine tract (PT) on the intron; U2AF1: binds the AG dinucleotide, interact with U2AF2 and SRSF2 and recruitment of ZRSR2 and U2AF1L4 RNA-binding proteins; U2 snRNP: recruitment of SF3A1, SF3B1, and SAP130 to 3′splicing site. U4/U6 and U5 complex: recruitment of U1 and LUC7L2 at 5’ splicing site; PRFP8: catalyzation the release of the intron lariat and ligation of exons on BP and PT regions. *SAP130: Official gene name is SF3B3.; **B** ATXN2 pre-mRNA variants (NCBI—Gene ID: 6311); **C** MAP2 pre-mRNA representative lenght of the 52 variants (NCB1—Gene ID: 4133); **D** Ataxin-2 variants mRNA expression; **E** MAP-2 mRNAs global variants expression. mRNA levels of were measured by RT-qPCR after 24h and 48h of incubation with 15 µM indacaterol, α-tubulin was used as house-keeping gene, results are expressed in fold change after being normalized to the corresponding untreated control cells. Statistical significance was determined by two-way ANOVA (mean ± 1 SD of three independent experiments (# *p* < 0.05; **p < 0.01; ***p < 0.001). UD: undertermined (not or low expressed)

In order to validate the inihibitory effect of indacaterol on SRSF6, the RNA expression of two splicing variants of one target gene of SRSF6 were evaluated. Indeed, a change in the expression or activity of SRSF6 would impact the proportion of the different RNA variants of the SRSF6 targeted RNAs. SRSF6 is implied in spliceosome recruitment on specific genes as described in Fig. 4-A [21]. Ataxin 2 (ATXN2) and microtubule binding protein 2 (MAP2) RNAs are known to be targeted by SRSF6 for the expression of alternative splicing variants.

ATXN2 presents 5 RNA variants (Fig. 4-B). Indacaterol induced a significant overexpression of ATXN-TOT (total ATXN RNA) at 24h, with a return to normal expression at 48h. Interestingly, only the variant 5 ATXN-V5 showed the same evolution in its expression. This return to normal expression can be explained by a cross regulation of ATXN2 variant expression by another splicing factor or to the loss of effect over time (Fig. 4-D).

MAP2 has 52 RNA variants. These variants present three major length of RNA: short, medium and long (Fig. 4-C). It was not possible to design primers for the long variants only, its expression has thus been deduced by substracting short and medium RNA expression from the total MAP2 RNA expression. Indacaterol induced the overexpression of MAP2-TOT (total MAP2 RNA) at 24h, which became significant at 48h. Data suggest that this was due to the overexpression of MAP2-medium variants (Fig. 4-E).

Cells exposed to indacaterol displayed a change in the proportion of the ATXN-V5 and of the MAP2-medium length RNA variants. These results indirectly validate the effective inhibition of SRSF6 by indacaterol.

**Fig. 5:**
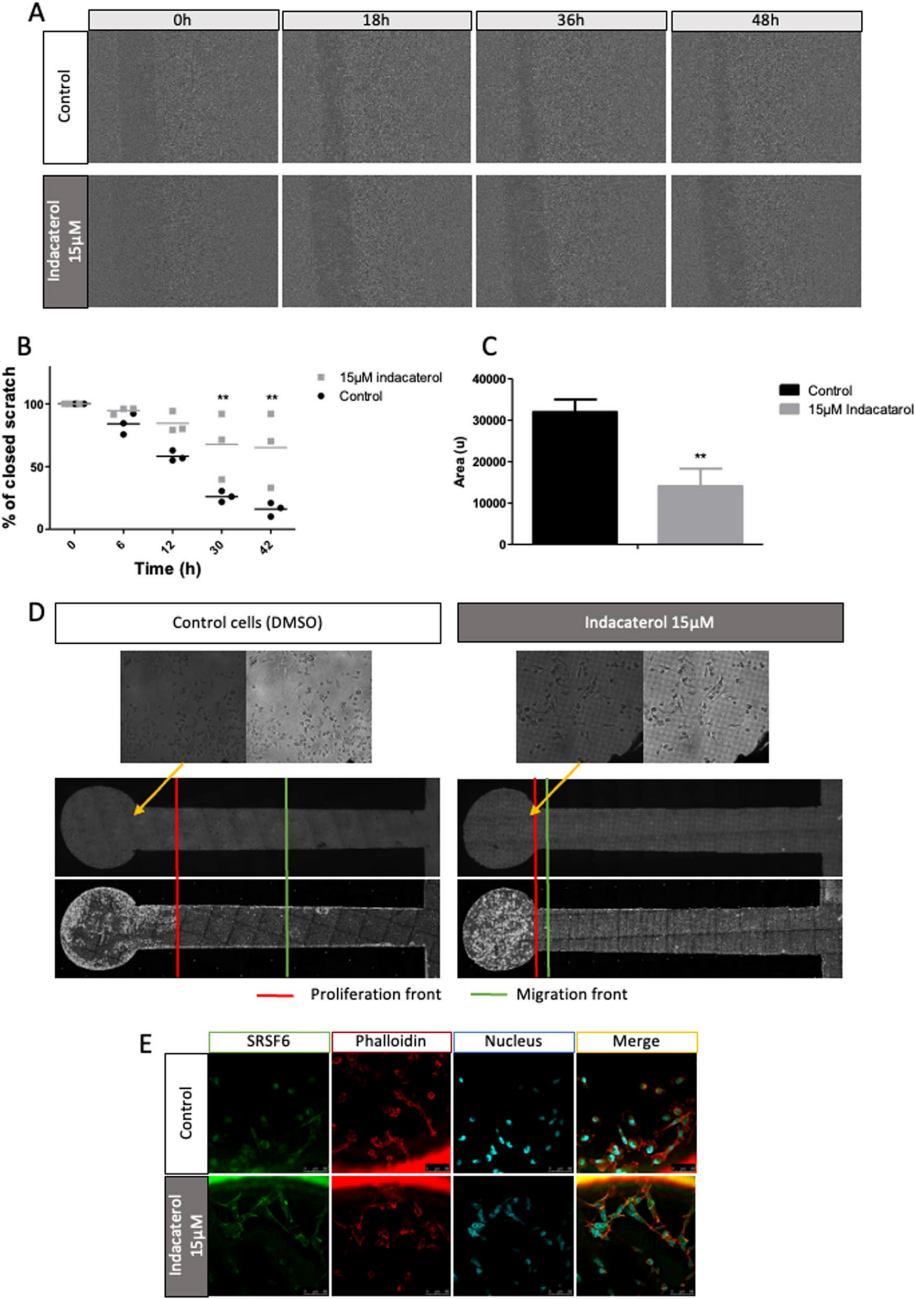
Effect of indacaterol on collective and IGDQ-induced MDA-MB-231 cell migration. MDA-MB-231 cells were incubated or not with 15 µM of indacaterol in DMSO. **A** *Cytonote* holographic wound healing time-lapse imaging for 48h on control and indacaterol incubated cells; **B** Represented as the percentage of closed scratch along time, statistical significance was determined by two-way ANOVA (mean ± 1 SD of three independent experiments) (*p < 0.05; **p < 0.01; ***p < 0.001); **C** Represented by cell relative speed (relative distance per hour), statistical significance was determined by unpaired t-test with Welch’s correction (mean ± 1 SD of three independent experiments) (*p < 0.05; **p < 0.01; ***p < 0.001). **D** *Ovizio* holographic single IGDQ-induced cell migration on engineered surfaces, non incubated or incubated with 15 µM of indacaterol for 4 days; **E** SRSF6 (green) and phalloïdin (cytoskeleton – red) and nucleus (UV – Hoechst) immunofluorescent labeling of MDA-MB-231 cells grown on IGDQ-exposing surfaces for 5 days

In order to study the impact of SRSF6 inhibition on cell migration, collective migration was evaluated using wound healing assays, in the presence of mitomycin C to inhibit cell proliferation (Fig. 5 – A, B and C). The control cells filled more than 85% of the wound in 48h while indacaterol incubated cells only filled 35% of the wound (Fig. 5 – A and B). A significative reduction by two fold of relative closing speed was observed in indacaterol-incubated cells compared to control cells (Fig. 5 – C). These results indicate that indacaterol inhibited collective cell migration.

To investigate the effect of indacaterol incubation on IGDQ-induced directional single cell migration, cells were seeded on the motogenic surfaces (Fig. 1) and the migration and proliferation of the cells were monitored by taking holographic images each day for 5 days (Fig. 5 – D). Normal proliferation was observed in both control and indacaterol-incubated cells. Migrating control cells, at sub-cm scale length, were observed along the IQDD gradient (Fig. 5 – D left). However, no migrating cells were observed in indacaterol-exposed cells (Fig. 5 – D right). The results showed that indacaterol inhibited the IGDQ-triggered single cell migration.

SRSF6 immunolabeling revealed no apparent modification of abundance or subcellular localization in cells seeded on IGDQ-engineered surfaces and exposed to indacaterol compared to control cells. However, it has to be noted that cells looked bigger and with a more fibroblast-like shape when incubated with indacaterol. This was already observed after 24h (Fig. 5 – D and E).

**Fig. 6:**
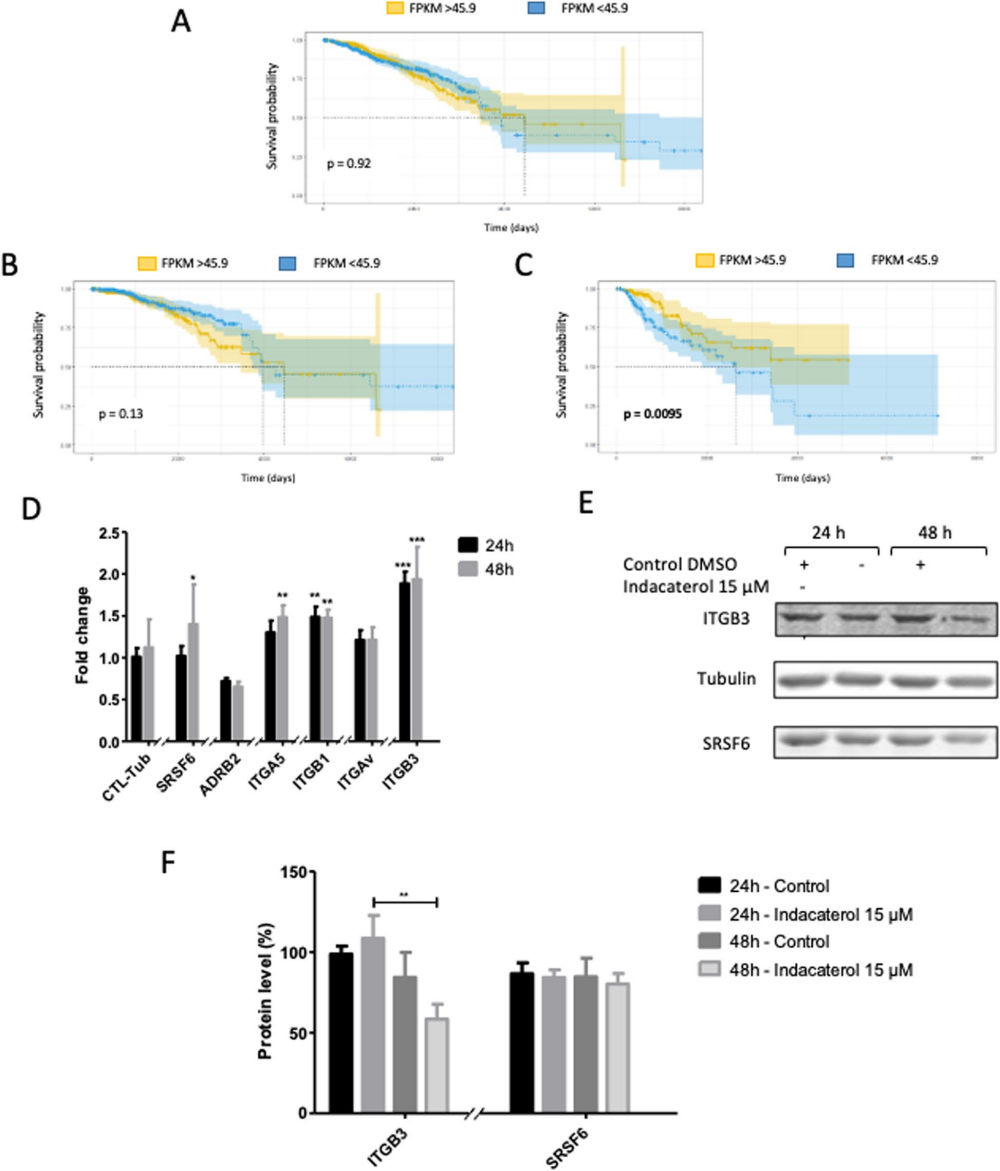
Indacaterol effect on SRSF6, ADRB2, integrin α5β1 and integrin αvβ3 expression in MDA-MB-231 cells. Kaplan–Meier plots presenting breast cancer patient survival in function of: **A** SRSF6 mRNA level, **B** SRSF6 mRNA level in low grades (I and II) and **C.** SRSF6 mRNA level in high grades (III and IV) from *“Protein atlas data”*; **D** mRNA levels of SRSF6, ADRB2, ITGA5, ITGB1, ITGAV and ITGB3 were measured by RT-qPCR after 24h and 48h of 15 µM indacaterol incubation, α-tubulin was used as house-keeping gene, results are expressed in fold change after being normalized to the corresponding untreated cells control; **E** Representative western-blot for SRSF6, ITGB3 and α-tubulin; **F** Quantification of protein abundance for SRSF6 and ITGB3 using α-tubulin charge as control (mean ± 1 SD of three independent experiments); Statistical significance was determined by two-way ANOVA (*p < 0.05; **p < 0.01; ***p < 0.001)

Using “the human protein atlas” database, survival curves related to SRSF6 RNA expression were calculated for breast cancer patients. Considering all grades (I to IV) or low grade only (I and II), the expression of SRSF6 did not impact the patient survival (Fig. 6 – A and B) [19]. However, when we took into account only patients with high grade breast cancer, a low expression of SRSF6 is related to a significant lower survival rate compared to high expression of SRSF6 (Fig. 6 – C). This is in opposition with its unfavorable prognostic marker status in renal and liver cancers where it is a high expression of SRSF6 which is related to a lower survival rate. This indicates a potential dual role of this protein depending on the cancer type.

### Indacaterol induced the upregulation of ITGB3 and ITGA5 mRNA

To evaluate the impact of indacaterol on the expression of genes of interest including genes whose expression may be impacted by this drug (ADRB2, ZO-1), their mRNA level was evaluated in cells incubated 24h and 48h in the presence of indacaterol.

Indacaterol induced a significant SRSF6 and ITGA5 mRNA overexpression at 48h and of ITGB1 and ITGB3 at 24 and 48h (Fig. 6 – D). At protein level, ITGB3 abundance was significantly reduced and no change for SRSF6 was observed after 48h of indacaterol incubation (Fig. 6 – E and F). A slight decrease in ADRB2 mRNA level was also observed at 24 and 48h in cells exposed to indacaterol (Fig. 6 – D). Regarding the other selected genes, no significant difference was observed in indacaterol-exposed cells except for ATXN2 at 24h (Additional file 2). Protein expression of ITGB3 and SRSF6 was evaluated, not showing significant difference in indacaterol-exposed cells compared to control ones (Fig. 6 – E and F). This suggests an indirect negative transcriptional regulation of SRSF6 and/or ADRB2 on ITGA5, ITGB3 and ITGB1 expression.

### Exposure to indacaterol induced endosomal and lysosomal vesicle dysregulation

**Fig. 7:**
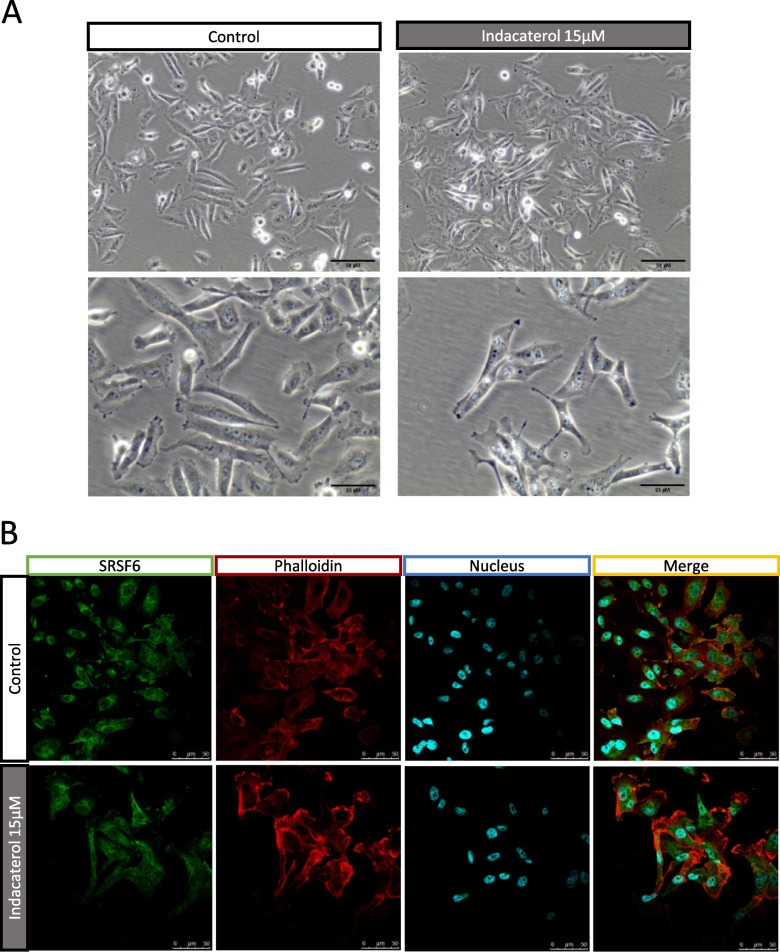
Effect of indacaterol on SRSF6 subcellular localization. **A** Optical imaging of MDA-MB-231 control cells and of 15 µM indacaterol incubated cells for 24h; **B** Immunofluorescence labeling of SRSF6 (green), cytoskeleton (phalloidin – alpha-tubulin – red) and nucleus (Hoechst – UV / blue) after 24h of indacaterol incubation

Optical microscopy pictures of MDA-MB-231 cells incubated with 15 µM of indacaterol showed undefined vesicles not present in control cells (Fig. 7 – A). SRSF6 labelling in cells cultured on classical culture plates confirmed the fibroblast-like shape induced by indacaterol (Fig. 7 – B).

**Fig. 8:**
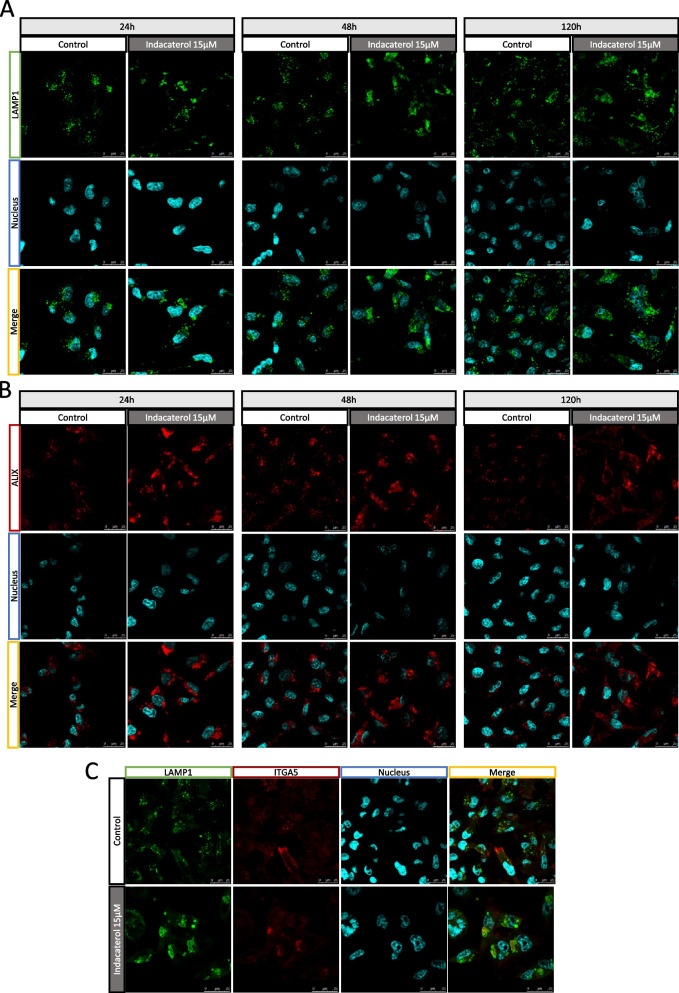
Effect of indacaterol on endosomes and lysosomes in MDA-MB-231 cells. **A** Immunofluorescence labeling of lysosomes (LAMP1 – green), and nucleus (Hoechst – UV / blue) after 24h, 48h or 120h of indacaterol incubation (15µM); **B** Immunofluorescence labeling of endosomes/multivesicular bodies (ALIX – red), and nucleus (Hoechst – UV / blue) after 24h, 48h or 120h of indacaterol incubation (15 µM); **C** Immunofluorescence labeling of lysosomes (LAMP1 – green), ITGA5 (red) and nucleus (Hoechst – UV / blue) after 24h of indacaterol incubation (15 µM)

In order to characterize these vesicles, cells incubated or not with indacaterol were labeled for lysosomal LAMP1 (Fig. 8 – A) and endosomal ALIX markers at 24h, 48h and 5 days (Fig. 8 – B). These large vesicles were positive for LAMP1 and ALIX staining and they were persistent at least for 5 days, while there were multiple small vesicles positive for both labeling in control cells (Fig. 8 A – B).

These results suggest a modification in both endosomal and lysosomal vesicle trafficking in cells exposed to indacaterol.

### Indacaterol induced ITGA5 sequestration in lysosomal vesicles

To futher investigate these large vesicles, integrin subcellular localization was also studied using confocal microscopy. A modification in ITGA5 labeling pattern was observed, with the apparition of ITGA5 aggregates in the cytoplasm and a decreased labeling at the membrane. Moreover, a co-labeling of ITGA5 with LAMP1 revealed that ITGA5 aggregates were found sequestered into lysosomes (Fig. 7 – C).

This suggests a modification of ITGA5 trafficking due to indacaterol exposure. This is combined with an abnormal lysosomal accumulation and a default in membrane addressing of ITGA5.

## Discussion

Understanding the mechanisms underlying cell migration is essential to find effective treatments against metastasis formation. ECM plays a major role all along this process. In this work, we focused on fibronectin type I mediated directional cell migration by triggering specific integrin beta 3 signaling pathway by IGDQ motif in an in vitro model using TNBC MDA-MB-231 cells [22]. To do so, we used a multi-omics methodology using i) RNAseq analysis of IGDQ-induced migrating cells compared to proliferating cells obtained on engineered surfaces and ii) proteomic data from a previous study obtained from cells invalidated for ITGB3 or ITGA5 compared to control cells. This permitted to determine an integrin / IGDQ-mediated migratory phenotype [4]. Using bioinformatics analyses, our studies allowed to highlight 10 targets which are linked to single cell IGDQ-mediated migration and integrin alpha 5 or integrin beta 3 signaling. Among them, SRSF6 turned out to be an interesting target for further study, in particular thanks to the possibility of directly inhibiting this protein using an FDA-approved drug: indacaterol [15]. Previous studies showed that indacaterol inhibited cell migration and invasion of fibrosarcoma cells with a reduction of TNFα – induced MMP-9 expression [23]. The activation of ADRB2 by isoproterenol was also described to be involved in the increase of the adhesion of fibroblasts and breast cancer cells on collagen and on fibronectin. However, unlike indacaterol which has an important specificity for ADBR2, isoproterenol also activates ADBR1 which induces cell contraction and therefore promotes cell migration [24].

**Fig. 9:**
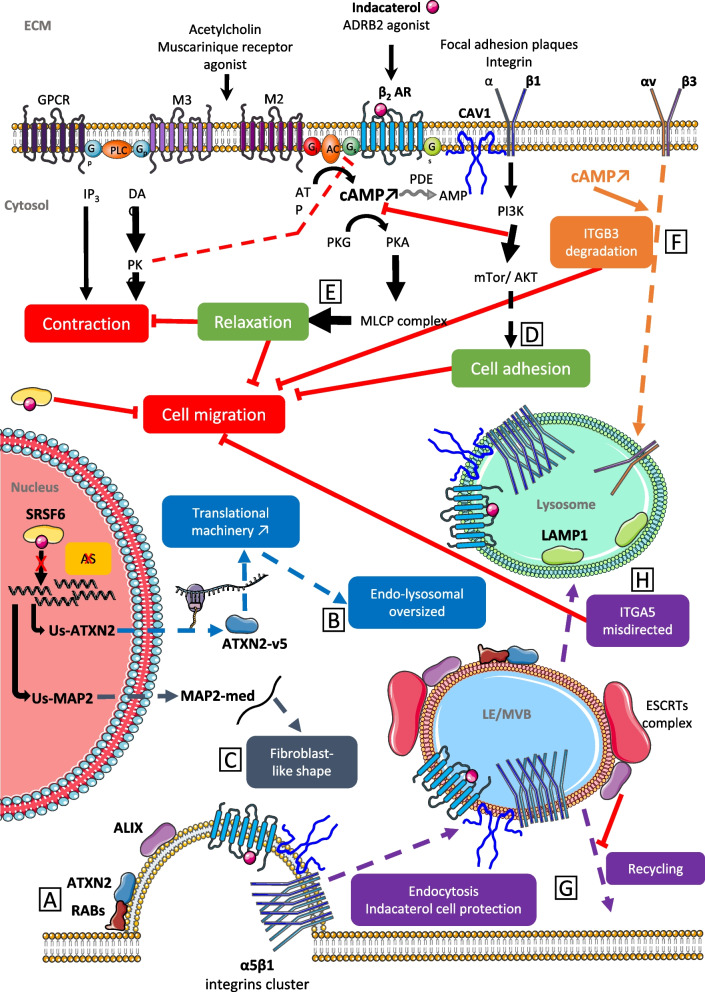
Schematic representation of putative mechanisms underlying indacaterol effects on MDA-MB-231 cell migration. Each impacted pathway is symbolized with a different color and a letter has also been assigned to help the explanation in the text. ADRB2/ β2 AR: adrenergic receptor β2; AKT: protein kinase B; ALIX: programmed cell death 6-interacting protein; AMP: adenosine monophosphate; AS: alternative splicing; ATP: adenosine triphosphate; ATXN2: ataxin 2; cAMP: cyclic adenosine monophosphate; CAV1: caveolin 1; DAG: diacylglycerol; ECM: extracellular matrix; ESCRTs:endosomal sorting complex required for transport; Gi: inhibitory G-protein; Gp: Gp protein alpha subunit; GPCR: G protein-coupled receptor; Gs: stimulatory G-protein; IP3: inositol trisphosphate; ITGA5: integrin alpha 5; ITGB3: integrin beta 3; LAMP1: lysosomal-associated membrane protein 1; LE: late endosome; M2: muscarinic receptors 2; M3: muscarinic receptors 3; MAP2: microtubule-associated protein 2; MLCP: myosin light-chain phosphatase; mTor: mechanistic target of rapamycin; MVB: multivesicular bodies; PDE: phosphodiesterase; PI3K: phosphoinositide 3-kinase; PKA: protein kinase A; PKC: protein kinase C; PKG: protein kinase G; RABs: Rab G-protein; SRSF6: splicing factor, arginine/serine-rich 6

The results presented here focused on the IGDQ Fn type I motif and on indacaterol impact on cell migration.We investigated the impact of indacaterol on cell migration using a triple negative breast cancer cell line. The collective migration was evaluated using wound-healing assay on uncoated surfaces while single cell migration was followed using surfaces exposing an IGDQ gradient. In this study, we showed that indacaterol blocked both collective and single cell IGDQ-mediated migration. It is important to notice that indacaterol can interact at least with two proteins: as an inhibitor of the splicing factor SRSF6 located at the nucleus and in cytoplasm, and as an agonist of the transmembrane adrenergic receptor beta 2 located at the plasma membrane. The later refers its role as a long-acting β2-agonist (LABA) commonly used as bronchodilator in chronic obstructive pulmonary disease (COPD) treatment [25]. To explain results that we obtained in this work, we analyzed both pathways targeted by this drug. Our hypothesis is schematically presented in Fig. 9.

Wan & al. recently determined the potential inhibitory effect on SRSF6 of indacaterol and its impact on cell migration in colorectal cancer cell model [15]. The impact of SRSF6 inhibition needs to be defined regarding the lack of knowledge on its mRNA targets or its other potential role as protein. In our model, SRSF6 is overexpressed in cells incubated with indacaterol suggesting a potential adaptation of the cells to compensate its inhibition. The implication of splicing factors SRSF4, SRSF6 and TRA2β was already demonstrated in cell proliferation and invasion promotion in normal mammary cells and in breast cancer cells [17]. Moreover, Park & al. demonstrated in MCF10 breast cancer cells overexpressing SRSF6 that ITGA5, ITGB2 and ITGB6 were overexpressed and alternative splicing variants of ITGB2, ITGB4, SRSF6 and ATXN2 were detected. ATXN2, one of our defined targets, was described to play different roles in autophagy and membrane protein (EGFR) traffic through interactions with Rabs GTPAses, in amytrophic lateral sclerosis (ALS). Moreover, ATXN2 was found to colocalize with Rab11 and Rab4 [26], which are proteins involved respectively in integrin α5β1 and αvβ3 recycling [27] (Fig. 9 – A). Furthermore, it was demonstrated that LPS-mediated stimulation of the Akt-mTOR pathway promoted the expansion of the endo-lysosome system, intracellular protein retention capacity and an increased activity of the translational machinery, showing the same vesicle pattern that the one observed in our study [28] (Fig. 9 – B). All together, this can partially explain the alterations observed in endocytosis and lysosomal vesicle sizes and in ITGA5 protein membrane addressing and trafficking regulation.

The microtubule binding protein 2 (MAP2) was found to be altered in Huntington disease (HD) due to its alternative splicing expression mediated by SRSF6 [29]. Indeed, high phosphorylated SRSF6 levels in neuronal cells of HD patients favored a specific MAP2 isoform and its localization at the stroma instead at the dendrites, with an alteration of the cytoskeleton with dendrite atrophy. SRSF6 indacaterol blockade can favor dendrite-like element formation and this may explain the increase in fibroblast-like shape observed in indacaterol incubated cells (Fig. 9 – C).

Indacaterol is also an ADRB2 agonist inducing an increase in cyclic adenosine monophosphate (AMPc). First, it was shown that direct or external stimulation of AMPc signaling, notably using ADRB2 agonist (isoproterenol), induced Epac-Rap1 dependent ITGB1-mediated fibronectin cell adhesion [30] (Fig. 9 – D). Secondly, in MDA-MB-231 breast cancer cells, it was shown that cAMP direct elevation reduced leptin-induced cell migration, via both cAMP/PKA- and cAMP-Epac-dependent pathways (Fig. 9 – E), and was related to a down-regulation of ITGB3 and FAK proteins level [31](Fig. 9 – F). Thirdly, it was demonstrated that high isoproterenol activation of ADRB2 receptor blocked ERK1/2-dependent cell proliferation while low isoproterenol favored Epac-dependent cell-adhesion coupled with ADRB2 localization in lipid raft. Integrin-mediated adhesion was described to involve lipid rafts in activated-integrin clusterization (LFA-1 and α4β1)-mediated in T-lymphocytes [32]. All together, these results suggest that the migration blockade phenotype obtained could be due to AMPc elevation, induced by indacaterol ADRB2 activation and to ITGB3 protein downregulation.

A crosstalk between ADRB2 and integrins, linked by caveolin-1 (CAV1) involved into vesicle formation and cargo caveolae-mediated endocytosis, was described during contraction signaling in airway smooth muscle cells [33]. Moreover, ADRB2 internalization was observed after cancer cell incubation with indacaterol [23] (Fig. 9 – G). ATXN2 is involved in late endosome/MVB (multivesicular bodies) and its loss in neuronal cells impaired endocytosis [34] (Fig. 9 – A). ALIX was also described to be involved into late endosome/MVB formation followed by their lysosome addressing, coupled with the silencing of internalized membrane receptors and their ligands (EGFR, GPCR) but not their recycling [35]. Taken together, this may explain the modified endosomes and lysosomes observed in indacaterol incubated cells and the accumulation of ITGA5 in lysosomes (Fig. 9 – H). We thus hypothesized that the mode of action of this drug can be related at least in part to alteration if the endo-lysosomal trafficking of integrins, preventing them to be exposed at the plasma membrane. Since we recently demonstrated that that ITGA5 knock-down reduced both collective and IGDQ-mediated breast cancer cell migration [13] this can explain how indacaterol inhibited cell migration.

Targeting ITGA5 to inhibit breast cancer cell migration has been recently achieved through different approaches with decreased metastasis capacity, thus demonstrating the potential of this approach for breast cancer therapy [21, 36, 37].

In this study, we highlight SRSF6 inhibition by indacaterol as a potential therapy to reduce cell migration involved in metastasis formation. The mode of action of this drug can be related to the endo-lysosomal trafficking regulation of integrins and ADRB2, the AMPc intracellular accumulation and SRSF6 alternative splicing regulation, which seemed to be central in integrin-dependent both collective and single IGDQ-induced cancer cell migration. Nowadays, membrane protein trafficking regulation and alternative splicing are considered as part of hallmarks in cancer [38, 39]. Further investigation to clarify the underlying molecular mechanisms is necessary. Our engineered surfaces allowed to highlight SRSF6 as being involved in integrin-mediated cell migration triggered by IGDQ. This work indicates indacaterol as a therapeutic candidate inhibitor of SRSF6 in TNBC to prevent cell migration and metastasis formation. Furthermore, since this study has been performed using MDA-MB-231 cells, it would also be interesting to study other cell lines, with different in vitro migration properties and/or different metastatic capacities when injected in vivo. In the future, it will be also interesting to investigate the other nine targets to see whether cell migration could be prevented in TNBC and other cancers.

## Materials and methods

### Cell culture

The metastatic breast cancer MDA-MB-231 cell line (pleural effusion – woman 51 year old—ATCC® HTB-26™ – *ATCC, Virginia, USA*) was cultivated in RPMI-1640 medium containing L-glutamate (*Gibco* – *Thermo Fisher Scientific, Massachusetts, USA*) supplemented with 10% fetal bovine serum (*Gibco* – *Thermo Fisher Scientific, Massachusetts, USA*). Cells were grown in a humidified atmosphere at 37°C with 5% CO_2_. Indacaterol maleate 15µM (stock solution at 20 mM in DMSO – *Sigma Aldrich / Merck, Germany*) was used to treat cells 24h after cell seeding, for 24h, 48h or 5 days.

### RNA extraction

MDA-MB-231 cells grown in T25 flask (0.4*10^6^ cells for 48h) were washed with PBS (20 mL phosphate buffer—KH_2_PO_4_ 0.5 mM at pH 7.4, 9 g NaCl qsp 1L bidistilled water) on ice, scraped off the plastic surface in PBS, centrifuged in 1.5 mL Eppendorf tube at 1000 rpm – 4°C and cell pellet harvested with 600 µL of RLT Lysis Buffer from *RNeasy Mini Kit* (*Qiagen, Germany*) in a 2 mL tube. Total RNA was extracted using the RNeasy Mini Kit and the automate *QIAcube* (*Qiagen, Germany*) under conditions “Large sample with DNase” and an elution volume of 30 μL. Total RNA concentration and quality were evaluated using *Nanodrop* N-100 spectrophotometer (*Isogen Life Science, Netherlands*) by measuring the absorbance at 260 nm and 230 nm. The samples were kept at -80°C.

### Reverse transcription quantitative PCR

Complementary DNA (cDNA) synthesis was performed from total RNA using *GoScript reverse transcription kit* (Promega), following the manufacturer instructions. 1 μg of total RNA was diluted with RNase-free water up to 12 μL. The samples were incubated at 70 °C for 5 min. A reaction mix containing 4 μL of GoScript buffer mix with random primers, 2 μL of GoScript enzyme mix and 2 μL of nuclease-free water was added to each sample. The samples underwent a temperature profile of 5 min at 25 °C, 60 min at 42 °C and 15 min at 70 °C. The cDNA sample tubes were stored at -20 °C.

Quantitative PCR (qPCR) was performed on a ViiA7 Treal-Time PCR System (Thermo Fisher Scientific) using *GoTaq G2®* qPCR kit (Promega). The qPCR mix was prepared with 5.56 μL of MilliQ water, 0.22 μL of each forward and reverse primer (Integrated DNA Technologies – to 300 nM (Additional table 3) and 10 μL *GoTaq G2®* qPCR Master Mix (Promega). 4 μL of 1/100 diluted cDNA and 16 μL of the reaction mix per well were added in a qPCR 96-well reaction plate (Thermo Fisher Scientific) and the plate was sealed and centrifuged at 600 rpm for 1 min. The temperature profile was 95 °C for 2 min followed by 40 cycles of amplification at 95 °C for 5 s, 60 °C for 20 s, and 70 °C for 20 s and a melting curve analysis at 65 °C to 95 °C with 0.5 °C per 5 s increments. The amplification was quantified using the threshold cycle (Ct) method using ViiA 7 Real-Time PCR software (Thermo Fisher Scientific).

Gene expression was determined using the ΔΔCt method with tubulin as the housekeeping gene and untreated control as the reference sample as followed:

$${\varvec{\Delta}}\mathbf{C}\mathbf{t} =\mathrm{ Ct\,gene\,of\,interest }-\mathrm{ Ct\,reference\,gene}$$

$${\varvec{\Delta}}{\varvec{\Delta}}\mathbf{C}\mathbf{t} =\mathrm{ \Delta\,Ct\,of\,the\,gene\,in\,condition\,of\,interest }-\mathrm{ \Delta \,Ct \,of \,the \,gene \,in \,the \,control \,condition}$$

$$\mathbf{F}\mathbf{o}\mathbf{l}\mathbf{d}\mathbf{c}\mathbf{h}\mathbf{a}\mathbf{n}\mathbf{g}\mathbf{e} = 2^{(-\mathrm{\Delta \Delta Ct })}=\mathrm{ relative \,gene \,expression \,compared \,to \,the \,control \,condition}$$

### Protein extraction

MDA-MB-231 cells grown in T25 flask (0.4*10^6^ cells for 48h) were washed with PBS on ice, scraped off the plastic surface in PBS, centrifuged in 1.5 mL Eppendorf tube at 1000 rpm – 4°C and cell pellet harvested with 30 µL of transmembrane protein lysis buffer (stock: 40 mM Trizma base, 150 mM KCl, 2 mM EDTA; lysis buffer: 1 mL stock lysis buffer, 30 µL Phosphatase Inhibitor Cocktail (PIC, Roche), 30 µL Phosphatase Inhibitor Buffer (PIB: 25 mM Na_3_VO_4_, 250 mM Para-NitroPhenyl Phosphate, 250 mM β-glycerophosphate, 125 nM NaF), 2 μL of β-mercaptoethanol 0.2%). After 15 min of incubation on ice, the samples were sonicated for 3 times 10 s and stored at -80°C.

### Western blotting

Quantification of proteins was carried out using Pierce 660 nm Protein assay (Thermo Fisher Scientific). Protein concentration were determined using the calibration curve (0 to 2 µg/µL bovine serum albumin). Equal protein loading (7 µg) was further confirmed by tubulin revelation.

Proteins were separated on 10% homemade polyacrylamide gel composed of 4% stacking gel (1.25 mL concentration buffer (0.5 M Tris, 0.4% sodium dodecylsulfate (SDS), pH 6.8), 0.5 mL acrylamide 30%, 2.25 mL distilled water, 50 μL ammonium persulphate (APS) 10%, 5 μL of tetramethylethylenediamine (TEMED)) and a 10% separating gel (1.2 mL staking buffer (1.5 M Tris, 0.4% SDS, pH 8.8), 1.7 mL acrylamide 30%, 2.1 mL of distilled water, 25 μL APS 10%, 5 μL of TEMED).

For each sample, 7 µg of proteins were mixed with 6 μL of loading blue 5X (10 mL concentration buffer, 10 mL SDS 20%, 5 mL of β-mercaptoethanol, 10 mL of glycerol, 17.5 mg of bromophenol blue, pH 6.8) and bidistilled water up to 30 μL, heated for 5 min at 100 °C and span briefly. 30 µL of each sample or 2 μL of molecular-weight size marker (New England Biolabs) were loaded in gel wells (7 µg protein diluted in bidistilled water). The migration was performed in a migration tank containing 1 L of running buffer 1X (2.5 mM Tris, 19.2 mM glycine, 5 mL SDS 20%) at 200 V, 400 mA and 15 W per gel, until the migration front reached the bottom of the running buffer gel.

Proteins were transferred from the gel onto a PVDF membrane (Bio-Rad), which was previously hydrated 1 min in methanol 100% and 10 min in transfer buffer (25 mM Tris, 150 mM glycin, 20% methanol, pH = 8.3 – Bio Rad). Liquid transfert method was used for 2 h at 70 V (Bio-Rad instruents) in cold condition.

Membranes were incubated with *Odyssey blocking buffer* (Li-Cor Biosciences) diluted 1/2 in PBS at room temperature for 1 h. The membranes were incubated overnight at 4°C with primary monoclonal antibodies (Additional Table 4) in blocking buffer completed with *Tween 20* (Bio-Rad) 0.1%. After rinsing with PBS – Tween 20 0.1% (5 min × 3), membranes were incubated with secondary antibodies (Additional Table 4) for 1h, rinsed with PBS (5 min × 3), dried and scan using *Odyssey imaging system* (Li-Cor Biosciences). Quantification of bands was made using *Odyssey software* and relative protein quantification was standardized to tubulin abundance.

### Immunofluorescence labeling

In 24 well plates (Corning) with uncoated or 1µg/cm^2^ fibronectin (Sigma) coated cover-slips (c.o.—Glaswarenfabrik Karl Hecht KG), 2.5 * 10^3^ MDA-MB-231 cells were seeded, in RPMI-1640 medium containing L-glutamate supplemented with 10% fetal bovine serum for 24h, 48h or 5 days. Cells were then fixed and permebabilized using methanol 80% / acetone 20% (stored at -20°C) for 10 min at RT (LAMP1 – ALIX) or 10 min PFA 4% fixation (0.04 g/mL – Merck), washed with PBS (3 × 10 min) and permeabilized 5 min with PBS-Triton 1% (*Triton X-100*—Carl Roth) (ITGA5 – SRSF6). Cells were washed with PBS-BSA 2% (Bovine serum albumin – VWR) and incubated with primary antibodies diluted in PBS-BSA 2% (Additional Table 4) overnight at 4°C in dark and humidified chamber. After being washed with PBS-BSA 2% (3 × 10 min), cells were incubated for 1 h at room temperature in dark with secondary antibodies, Hoechst (#H-21491 -Thermo Fisher Scientific) and probe (Additional Table 3). Cells were washed in PBS-BSA 2% and in PBS (2 × 5 min) and cover slips were mounted on microscope slides (VWR) with Mowiol mounting solution (Sigma-Aldrich) prewarmed at 56°C. Slides were kept at 4°C protected from light before the observation with the confocal laser scanning fluorescence microscope TCS SP5 (Leica).

### Wound healing assay

In 35mm x 10mm polystyrene cell culture dish (Corning), 0.8 * 10^6^ MDA-MB-231 cells were seeded for 24h at 37°C – 5% CO_2_, in in RPMI-1640 medium containing L-glutamate supplemented with 10% fetal bovine serum. The confluent cell layer was scraped to form an ~ 1 mm width wound. Fresh complete medium supplemented with 10 µg/mL mitomycin C (Sigma-Aldrich) was added on the scraped cells in order to inhibit cell proliferation. Live imaging in incubator at 37°C – 10% CO_2_ was monitored using Cytonote holographic system (Iprasense – *Horus* software), taking pictures every 20 min during 48h. Relative speed of migration and closing areas were determined using *ImageJ – Phase Wound Macro*.

### Peptide-associated cell migration along engineered surfaces

Gold surfaces (2 nm Ti, 10 nm Au on glass coverslips) were produced by physical vapor deposition at the LARN (UNamur) using plasma deposition chamber (*ATC-Orion 5 UHV with Load-Lock* – AJA International Inc.) and then were cleaned with UV – Ozone (2h—organic compounds removing). Peptides were coated (Au-SH interactions – Fig. 1 – A and B) to form engineered surfaces: a self-assembled monolayer (SAM) composed by an motogenic IGDQ gradient on 2D surface, filled by inert backfiller tetraethylenglycol (PS – Fig. 1 – A and B). After ethanol sterilization, engineered surfaces were placed into 24 well plates and a 0.6 µL drop containing cells at 1*10^6^ cells/mL were seeded at the lower concentration of IGDQ peptide gradient and filled after 4 min with RPMI-1640 medium containing L-glutamate supplemented with 10% fetal bovine serum. At day 2, cells were incubated with 15 µM of indacaterol solubilized into DMSO or with DMSO alone. Cell migration was monitored using Ovizio v1.0 holographic microscopy (*Ovizio Imaging System* – OsOne v5.1) once a day for 5 days (Fig. 4 – D). Electron microscopy images were also taken at day 5 (Fig. 1 – C and D).

### RNA sequencing analysis

#### Cells isolation

This experiement was done on 4 biological replicates and 16 engineered surfaces were used for each biological replicate to obtain enough migrating cells for the RNa sequencing. Cells were detached, using trypsin – 0.5 EDTA (*Gibco* – *Thermo Fisher Scientific, Massachusetts, USA*), from engineered surfaces after 5 days, taking into account their status: A. proliferating cells (Fig. 1 – C); B. late migrating cells and C. migrating cells (Fig. 1 – D). Cells were pelleted and resuspended in 7 µL of PBS in *Eppendorf® DNA LoBind tubes* (Merck).

#### Whole transcriptome amplification (WTA)

The sequencing librairy was produced following manufacturer instructions, using *QIAseq FX single cell RNA library kit* (Qiagen) which is compatible for low input samples (100 to 1000 cells). This technique permits to perform all steps in one tube.). First, cells were lysed and genomic DNA (gDNA) was degraded. A reverse-transcription was performed using random primers to have all RNAs, no polyadenylated RNAs enrichment because of the low number of starting cells. A concatemer was then formed with the cDNA obtained. These long cDNA templates are amplified using the ultra-high fidelity phi29 polymerase (WTA). the samples were stored at -20°C. A quality control (QC) was done using the PicoGreen (Invitrogen) for the quantification (Fluorescent intercalant) and the Bioanalyzer 2100 (Agilent) for the quality of cDNA cancatemer fragments. Those analysis were made at the Genomic core facility of GIGA – Liège.

#### Illumina library

The cDNA was enzymatically fragmented, starting from 500 ng for each sample. This step permitted to obtain fragments with a suitable size for Illumina sequencing technology (from 300 to 500 base pair (bp)). The adaptors, which contained common adaptors, a barcode specific for each sample and common sequencing primers, were ligated at the end of the inserts to obtain a paired-end library. Each library was then purified using magnetic beads and quantified using Illumina standard method. Libraries were stored at -20°C.

#### Sequencing

Libraries were quality controlled, quantified and pooled following *Illumina* instructions. To obtain RNA level experession, a single-read 50 bp (SR50) and 60X depth was performed on *HiSeq 2500* (Illumina), using manufacturer products and protocol, at GIGA’s genomic platform (Liège – Belgium).

#### Raw data analysis

HiSeq 2500 instrument generated sequencing raw data file (.bcl) and directly transformed it into FastQ binary files (.FASTA), containing all rads sequence, their barcode attribution and their quality (*HiSeqFastQ* tool). From this step, one file for each sample has been obtained and treated separately. Read sequences from FastQ files were mapped against the human reference genome GRCh37/hg19 (http://hgdownload.soe.ucsc.edu/downloads.html#human) using *TopHat Alignment v1.1.0 Illumina* tool via *TopHat 2* aligner, given binary SAM files [40]. To reduce their size, SAM files were converted into compressed BAM files and its corresponding BAI file, needed to read BAM files. BAM/BAI files were produced at GIGA’s genomic platform (Liège – Belgium). Two samples failed the QC and were removed form the following analysis (one from A and one from C samples). Heatmap representing RNAseq global gene expression for each replicate (N) and their segregation by similarity permitted to validate that they are similar for a defined phenotype (A: static/proliferating phenotype, B: late migrating phenotype or C: migrating phenotype – Additional file 3).

#### Differential expression (DE) analysis

Followed analysis were made at URBC – UNamur (Belgium).

Count of the reads (.COUNT or RPKM) was generated for each sample. Then a differential expression (DE) was done: the results of the four or three replicates from one condition were merged and conditions were compared two by two, using specific statistical analysis, to obtain ranked differentially expressed genes. This step permitted to compare two conditions regarding the count obtained for each transcript and to identify the genes which were differentially expressed. Illumina tools were used to obtain both COUNT and DE files. To reinforce the results, two analysis strategies were used to obtain both DE for each comparison (A vs B, A vs C and B vs C) and RNA (reads) expressed count for each sample, using different tools and statistical analysis. *RNA Expresse v1.1.0* Illumina tool permited to obtain the read alignement (BAM/BAI files) with their gene assignement (.COUNT files) using *STAR* aligner and the DEs using *DESeq2* [41, 42]. RNA-Seq Differential Expression v1.0.0 Illumina tool permitted to obtain the COUNT files and DEs using *DESeq2* [42]. A false discovery rate (FDR) or p-value under 0.1 was used as threshold to select significant differentially expressed genes.

#### Gene ontology (GO) analysis

GO analysis was done using *Gene Set Enrichment Analysis* (functionnal enrichment analysis – GSEA – used on 2020–01) software, starting from.COUNT files obtained with both illumina tools. It permitted to compare conditions two by two, using corresponding replicates, and to determine statistically and concordant differences between them, with a comparison to an a priori set of genes. This analysis permited to obtain a phenotypical point of view of RNA differential expression. For the functional enrichement analysis, the “c5” GO set was used, including three type of sets: molecular function (MF), cellular component (CC) and biological process (BP).

#### Multiomic analysis

BP obtained for RNA sequencing presented and protemic data obtained previously obtained in our lab were used for the multiomic analysis. From the common GO-BP obtained, diffenrentially expressed genes were obtained by comparison with corresponding gene sets (Fig. 2).

### Statistics

Data from mRNA, protein and cell migration are presented as mean ± SD. Two-way ANOVA was performed followed by the post-hoc Bonferroni test. Data from relative migration speed was analyzed using an unpaired t-test with Welch’s correction. Significant p-value are presented as follow: *p* < 0.05, **p < 0.01 and ***p < 0.001 (*GraphPad Prism 5 v.5.01*).

### Omics raw data availability

Data from the RNA sequencing are available on the repository Annotare/ Array express of EBI (https://www.ebi.ac.uk/biostudies/ArrayExpress/studies) on the accession number E-MTAB-10477.

Data from the proteomic analysis are available on the repository PRIDE, RRID:SCR_003411.

### Supplementary Information

**Additional file 1.**

## References

[1] Bareche Y, Venet D, Ignatiadis M, Aftimos P, Piccart M, Rothe F, Sotiriou C (2018). Unravelling triple-negative breast cancer molecular heterogeneity using an integrative multiomic analysis. Ann Oncol.

[2] Häger A, Alexander S, Friedl PH. Cancer invasion and resistance. EJC Suppl. EJC Off. J. EORTC, Eur. Organ. Res. Treat. Cancer ... [et al.] 2013;11, 291–293. 10.1016/j.ejcsup.2013.07.055. 10.1016/j.ejcsup.2013.07.055 PMC4041189 26217149

[3] Porquet N, Gout S, and Huot J. The Metastatic Process: An Overview. In, 2010. pp. 1–31. 10.1007/978-90-481-8833-8_1.

[4] Corvaglia V, Marega R, De Leo F, Michiels C, Bonifazi D (2016). Unleashing cancer cells on surfaces exposing motogenic IGDQ peptides. Small.

[5] Hynes RO (1986). Fibronectins. Sci Am.

[6] Georges-Labouesse EN, George EL, Rayburn H, Hynes RO (1996). Mesodermal development in mouse embryos mutant for fibronectin. Dev Dyn.

[7] Astrof S, Hynes RO (2009). Fibronectins in vascular morphogenesis. Angiogenesis.

[8] Lin TC, Yang CH, Cheng LH, Chang WT, Lin YR, Cheng HC (2019). Fibronectin in Cancer: Friend or Foe. Cells.

[9] von Au A, Vasel M, Kraft S, Sens C, Hackl N, Marx A, Stroebel P, Hennenlotter J, Todenhöfer T, Stenzl A (2013). Circulating fibronectin controls tumor growth. Neoplasia (United States).

[10] Mongiat M, Andreuzzi E, Tarticchio G, Paulitti A (2016). Extracellular matrix, a hard player in angiogenesis. Int J Mol Sci.

[11] White DP, Caswell PT, Norman JC (2007). αvβ3 and α5β1 integrin recycling pathways dictate downstream Rho kinase signaling to regulate persistent cell migration. J Cell Biol.

[12] Missirlis D, Haraszti T, Scheele CVC, Wiegand T, Diaz C, Neubauer S, Rechenmacher F, Kessler H, Spatz JP (2016). Substrate engagement of integrins α5 β1 and αv β3 is necessary, but not sufficient, for high directional persistence in migration on fibronectin. Sci Rep.

[13] Ayama-Canden S, Tondo R, Piñeros L, Ninane N, Demazy C, Dieu M, Fattaccioli A, Tabarrant T, Lucas S, Bonifazi D (2022). IGDQ motogenic peptide gradient induces directional cell migration through integrin (αv)β3 activation in MDA-MB-231 metastatic breast cancer cells. Neoplasia.

[14] Schor SL, Ellis I, Banyard J, Schor AM (1999). Motogenic activity of IGD-containing synthetic peptides. J Cell Sci.

[15] Wan L, Yu W, Shen E, Sun W, Liu Y, Kong J, Wu Y, Han F, Zhang L, Yu T (2019). SRSF6-regulated alternative splicing that promotes tumour progression offers a therapy target for colorectal cancer. Gut.

[16] Black DL (2003). Mechanisms of alternative pre-messenger RNA splicing. Annu Rev Biochem.

[17] Park SH, Brugiolo M, Akerman M, Das S, Urbanski L, Geier A, Kesarwani AK, Fan M, Leclair N, Lin KT (2019). Differential functions of splicing factors in mammary transformation and breast cancer metastasis. Cell Rep.

[18] Moradpoor R, Gharebaghian A, Shahi F, Mousavi A, Salari S, Akbari ME, Ajdari S, Salimi M (2020). Identification and validation of stage-associated pbmc biomarkers in breast cancer using MS-based proteomics. Front Oncol.

[19] Uhlen M, Zhang C, Lee S, Sjöstedt E, Fagerberg L, Bidkhori G, Benfeitas R, Arif M, Liu Z, Edfors F (2017). A pathology atlas of the human cancer transcriptome. Science (80-. ).

[20] She W, Shao J, Jia R (2021). Targeting splicing factor SRSF6 for cancer therapy. Front Cell Dev Biol.

[21] Visconte V, Nakashima MO, Rogers HJ (2019). Mutations in splicing factor genes in myeloid malignancies: Significance and impact on clinical features. Cancers (Basel).

[22] Kiosses WB, Shattil SJ, Pampori N, Schwartz MA (2001). Rac recuits high-affinity integrin αvβ3 to lamellipodia in endothelial cell migration. Nat Cell Biol.

[23] Lee SU, Ahn KS, Sung MH, Park JW, Ryu HW, Lee HJ, Hong ST, Oh SR (2014). Indacaterol inhibits tumor cell invasiveness and MMP-9 expression by suppressing IKK/NF- kB activation. Mol Cells.

[24] Boulay G, Malaquin N, Loison I, Foveau B, Van Rechem C, Rood BR, Pourtier A, Leprince D (2012). Loss of Hypermethylated in Cancer 1 (HIC1) in breast cancer cells contributes to stress-induced migration and invasion through β-2 adrenergic receptor (ADRB2) misregulation. J Biol Chem.

[25] Dahl R, Chung KF, Buhl R, Magnussen H, Nonikov V, Jack D, Bleasdale P, Owen R, Higgins M, Kramer B (2010). Efficacy of a new once-daily long-acting inhaled β2-agonist indacaterol versus twice-daily formoterol in COPD. Thorax.

[26] Farg MA, Sundaramoorthy V, Sultana JM, Yang S, Atkinson RAK, Levina V, Halloran MA, Gleeson PA, Blair IP, Soo KY (2014). C9ORF72, implicated in amytrophic lateral sclerosis and frontotemporal dementia, regulates endosomal trafficking. Hum Mol Genet.

[27] de Franceschi N, Hamidi H, Alanko J, Sahgal P, Ivaska J (2015). Integrin traffic-the update. J Cell Sci.

[28] Hipolito VE, Diaz JA, Tandoc KV, Oertlin C, Ristau J, Chauhan N, Saric A, Mclaughlan S, Larsson O, Topisirovic I (2019). Enhanced translation expands the endo-lysosome size and promotes antigen presentation during phagocyte activation. PLoS Biol.

[29] Fernández-Nogales M, Santos-Galindo M, Hernández IH, Cabrera JR, Lucas JJ (2016). Faulty splicing and cytoskeleton abnormalities in Huntington’s disease. Brain Pathol.

[30] Rangarajan S, Enserink JM, Kuiperij HB, De Rooij J, Price LS, Schwede F, Bos JL (2003). Cyclic AMP induces integrin-mediated cell adhesion through Epac and Rap1 upon stimulation of the β2-adrenergic receptor. J Cell Biol.

[31] Spina A, Di Maiolo F, Esposito A, Sapio L, Chiosi E, Sorvillo L, Naviglio S (2012). CAMP elevation down-regulates β3 integrin and focal adhesion kinase and inhibits leptin-induced migration of MDA-MB-231 breast cancer cells. Biores Open Access.

[32] Bruzzone A, Saulière A, Finana F, Sénard J, Lüthy I, Galés C (2014). Dosage-dependent regulation of cell proliferation and adhesion through dual β _2_ -adrenergic receptor/cAMP signals. FASEB J.

[33] Lajoie P, Nabi IR (2010). Lipid rafts, caveolae, and their endocytosis. Int Rev Cell Mol Biol.

[34] Tang BL (2016). C9orf72’s interaction with rab GTPases—modulation of membrane traffic and autophagy. Front Cell Neurosci.

[35] Sun S, Zhou X, Zhang W, Gallick GE, Kuang J (2015). Unravelling the pivotal role of Alix in MVB sorting and silencing of the activated EGFR. Biochem J.

[36] Limam I, Abdelkarim M, El Ayeb M, Crepin M, Marrakchi N, Di Benedetto M (2023). Disintegrin-like Protein Strategy to Inhibit Aggressive Triple-Negative Breast Cancer. Int J Mol Sci.

[37] Desjardins-Lecavalier N, Annis MG, Nowakowski A, Kiepas A, Binan L, Roy J, Modica G, Hébert S, Kleinman CL, Siegel PM (2023). Migration speed of captured breast cancer subpopulations correlates with metastatic fitness. J Cell Sci.

[38] Wang X, Li S (2014). Protein mislocalization: Mechanisms, functions and clinical applications in cancer. Biochim Biophys Acta - Rev Cancer.

[39] Oltean S, Bates DO (2014). Hallmarks of alternative splicing in cancer. Oncogene.

[40] Kim D, Pertea G, Trapnell C, Pimentel H, Kelley R, Salzberg SL (2013). TopHat2: accurate alignment of transcriptomes in the presence of insertions, deletions and gene fusions. Genome Biol.

[41] Dobin A, Davis CA, Schlesinger F, Drenkow J, Zaleski C, Jha S, Batut P, Chaisson M, Gingeras TR (2013). STAR: ultrafast universal RNA-seq aligner. Bioinformatics.

[42] Anders S, Huber W (2010). Differential expression analysis for sequence count data. Genome Biol.

