# Supplementary material for: Indacaterol inhibits collective cell migration and IGDQ-mediated single cell migration in metastatic breast cancer MDA-MB-231 cells
Source: Cell Commun Signal. 2023 Oct 30;21:301. doi: 10.1186/s12964-023-01340-9 (PMC10614342; doi:10.1186/s12964-023-01340-9)
Supplement: Supplementary file 2 — Additional file 1. [file 12964_2023_1340_MOESM1_ESM.pdf]

Tittle

Indacaterol inhibits collective cell migration and IGDQ-mediated single cell migration in metastatic breast cancer MDA-MB-231 cells

Authors

Sophie AYAMA-CANDEN, Rodolfo TONDO, Martha Liliana PINEROS LEYTON, Noëlle NINANE, Catherine DEMAZY, Marc DIEU, Antoine FATTACCIOLI, Aude SAUVAGE, Tijani TABARRANT, Stéphane LUCAS, Davide BONIFAZI, Carine MICHIELS

Additional files

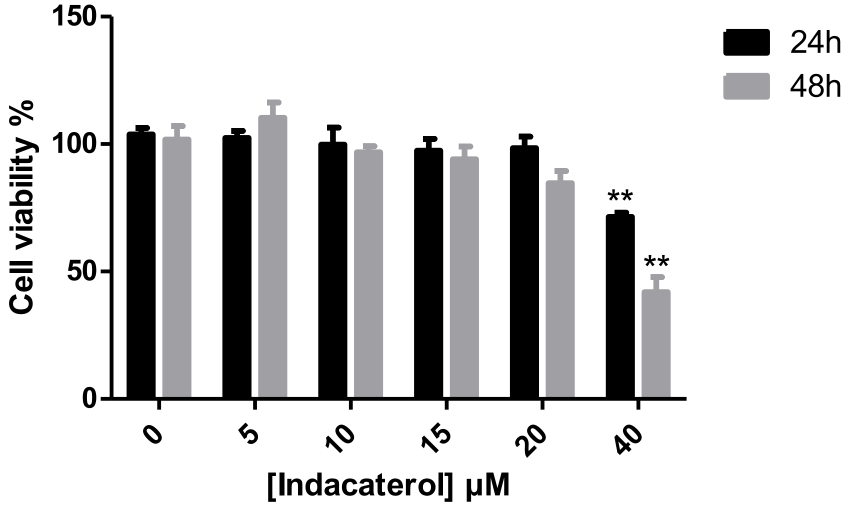

Additional file 1: Cell viability of MDA-MB-231 cells incubated with 15 µM of indacaterol.

MTT cytotoxicity assay using 0 to 40 µM of indacaterol for 24h or 48h. Statistical significance was determined by two-way ANOVA (mean ± 1 SD of three independent experiments) **$p < 0.01$).

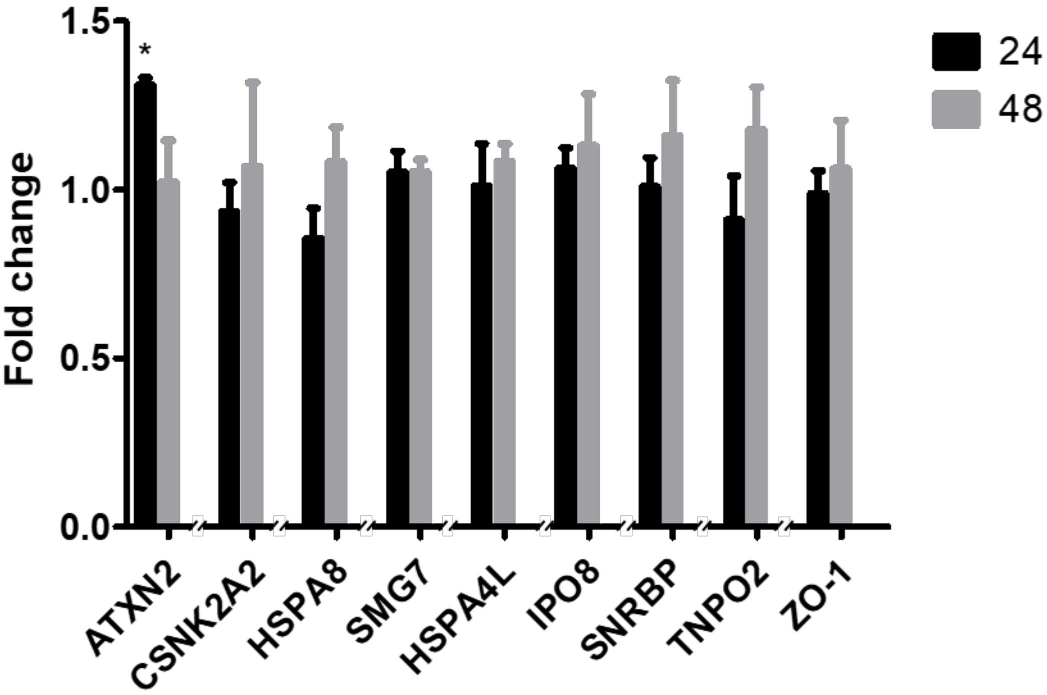

Additional file 2: mRNA levels of genes of interest, obtained from the multi-omic analysis, in MDA-MB-231 cells incubated or not with indacaterol.

mRNA levels were measured by RT-qPCR after 24h and 48h of 15 µM indacaterol incubation, α-tubulin was used as house-keeping gene (CTL-Tub), results are expressed in fold change after being normalized to the untreated control cells. Statistical significance was determined by two-way ANOVA (mean ± 1 SD of three independent experiments) (*$p < 0.05$).

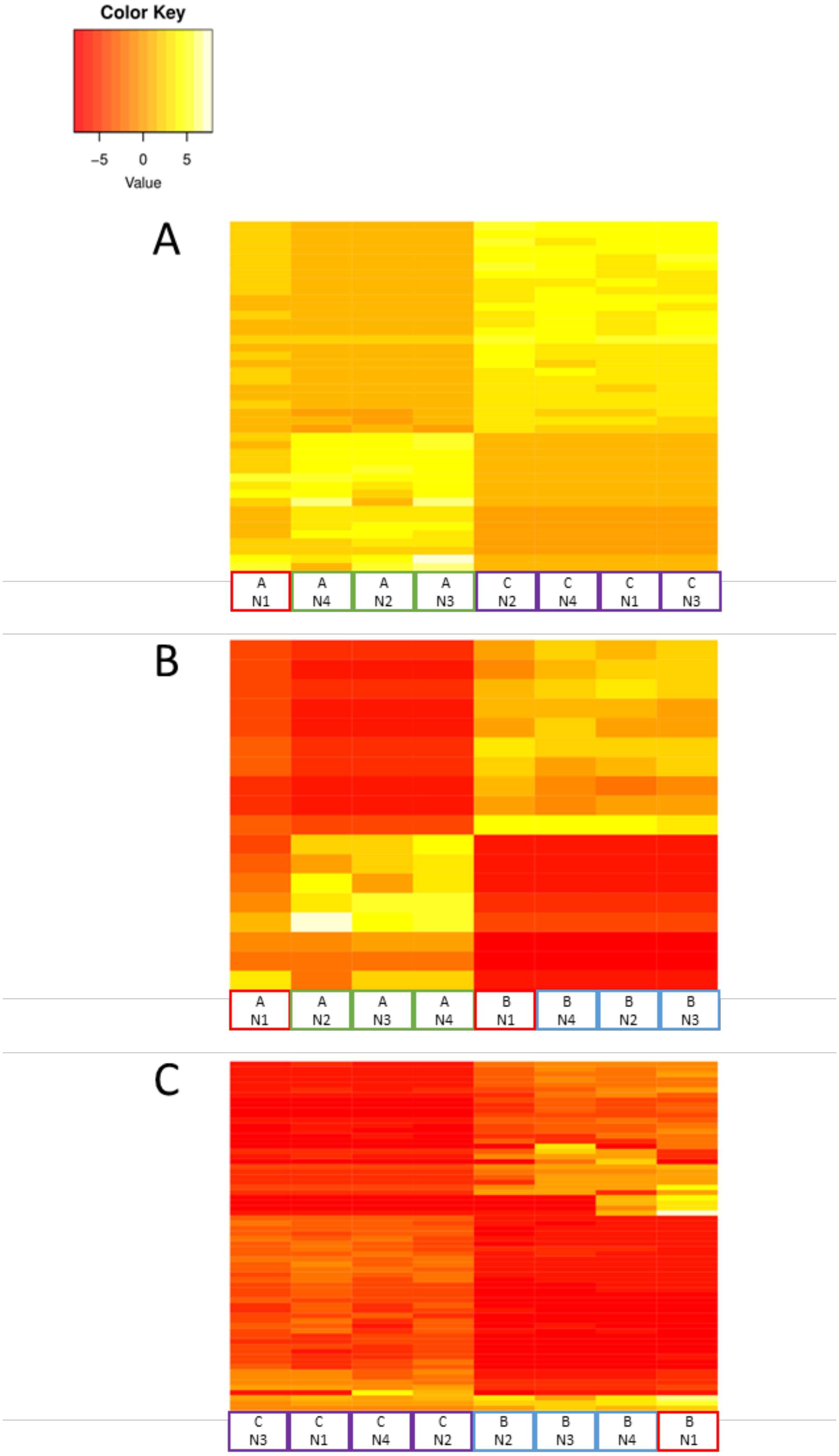

Additional file 3: Heatmap representing RNAseq global gene expression for each replicate (N) and their segregation by similarity. **A:** static/proliferating phenotype; **B:** late migrating phenotype; **C:** migrating phenotype.

Additional table 1: Target gene counts, p-value and Log2 fold change obtained from RNAseq data analysis using **A.** Differential expression tool (Illumina), **B.** RNA-Express tool (Illumina) and from proteomic data using **C.** Scaffold tool. Significant p-values are in bold, gene/protein upregulated in green and downregulated in red.

A Gene name	Gene symbol	Count - RNAExpress			p-value - RNAExpress			Log2 Fold change - RNAExpress		
		A	B	C	A vs B	A vs C	B vs C	A vs B	A vs C	B vs C
Ataxin 2	ATXN2	59,33	68,00	48,75	0,785	0,587	0,274	0,11	-0,20	-0,36
Casein kinase 2 alpha 2	CSNK2A2	34,00	13,67	21,75	**0,029**	0,285	0,185	**-0,93**	-0,41	0,53
Heat shock protein family A (Hsp70) member 8	HSPA8	68,67	82,00	50,25	0,631	0,205	**0,057**	0,18	-0,40	**-0,61**
RNA binding fox-1 homolog 1	RBFOX1	37,67	56,33	39,25	0,428	0,953	0,313	0,34	-0,02	-0,39
SMG7 nonsense mediated mRNA decay factor	SMG7	70,67	97,00	90,50	0,474	0,556	0,813	0,29	0,21	-0,08
**Protein name**	**Protein symbol**	Count - RNAExpress			p-value - RNAExpress			Log2 Fold change - RNAExpress		
		A	B	C	A vs B	A vs C	B vs C	A vs B	A vs C	B vs C
Casein kinase 2 alpha 2	CSNK2A2	34,00	13,67	21,75	**0,029**	0,285	0,185	**-0,93**	-0,41	0,53
Heat shock protein family A (Hsp70) member 4 like	HSPA4L	50,00	49,67	41,50	0,797	0,506	0,617	-0,11	-0,25	-0,17
Importin 8	IPO8	79,00	86,67	74,25	0,959	0,781	0,705	0,02	-0,10	-0,13
Small nuclear ribonucleoprotein polypeptides B and B1	SNRPB	21,00	24,00	13,75	0,881	0,431	0,268	0,07	-0,31	-0,46
Serine and arginine rich splicing factor 6	SRSF6	74,33	70,67	71,75	0,741	0,757	0,911	-0,13	-0,10	0,03
Transportin 2	TNPO2	63,33	35,00	98,75	0,261	0,216	**0,005**	-0,49	0,49	**1,12E+14**

B Gene name	Gene symbol	Count - DE			p-value - DE			Log2 Fold change - DE		
		A	B	C	A vs B	A vs C	B vs C	A vs B	A vs C	B vs C
Ataxin 2	ATXN2	27,541	33,775	12,755	**0,001**	**0,002**	0,996	**7,14E+14**	**6,39E+14**	-0,75
Casein kinase 2 alpha 2	CSNK2A2	30,877	25,910	34,546	1,000	1,000	1,000	-1,42E+14	-8,67E-01	0,55
Heat shock protein family A (Hsp70) member 8	HSPA8	44,458	33,452	45,453	1,000	**0,001**	0,996	-1,35E+14	**-8,01E+14**	-6,66E+14
RNA binding fox-1 homolog 1	RBFOX1	27,107	23,527	20,964	NaN	0,845	**0,001**	-3,47E+14	3,07E+14	**6,62E+13**
SMG7 nonsense mediated mRNA decay factor	SMG7	36,503	42,291	43,726	1,000	**0,001**	1,000	6,71E+14	**7,42E+14**	0,71
**Protein name**	**Protein symbol**	Count - DE			p-value - DE			Log2 Fold change - DE		
		A	B	C	A vs B	A vs C	B vs C	A vs B	A vs C	B vs C
Casein kinase 2 alpha 2	CSNK2A2	30,877	25,910	34,546	1,000	1,000	1,000	-1,42E+14	-0,87	0,55
Heat shock protein family A (Hsp70) member 4 like	HSPA4L	53,882	29,542	15,962	Outlier	0,721	0,832	Outlier	6,31E+14	6,04E+14
Importin 8	IPO8	51,815	23,270	27,460	0,802	1,000	1,000	0,25	0,36	0,10
Small nuclear ribonucleoprotein polypeptides B and B1	SNRPB	14,270	17,985	38,447	0,952	1,000	1,000	0,20	-0,33	-0,51
Serine and arginine rich splicing factor 6	SRSF6	64,027	70,601	44,353	0,799	NaN	0,820	0,85	-7,23E+14	-8,06E+14
Transportin 2	TNPO2	31,617	47,419	44,273	0,668	1,000	1,000	-0,73	-0,28	0,45

C Gene name	Gene symbol	Spectral count - Proteomic			p-value - Proteomic		Fold change - Proteomic		Log2 Fold change - Proteomic	
		shCTL	shITGA5	shITGB3	shITGA5	shITGB3	shITGA5	shITGB3	shITGA5	shITGB3
Ataxin 2	ATXN2	4,00	3,67	4,67	0,990	0,670	1,00	1,20	0,00	0,26
Casein kinase 2 alpha 2	CSNK2A2	5,67	3,00	3,33	**0,031**	**0,054**	0,60	0,60	**-0,74**	**-0,74**
Heat shock protein family A (Hsp70) member 8	HSPA8	300,67	310,33	302,00	0,150	0,720	1,10	1,00	0,14	0,00
RNA binding fox-1 homolog 1	RBFOX1	*NA*	*NA*	*NA*	*NA*	*NA*	*NA*	*NA*	*NA*	*NA*
SMG7 nonsense mediated mRNA decay factor	SMG7	*NA*	*NA*	*NA*	*NA*	*NA*	*NA*	*NA*	*NA*	*NA*
**Protein name**	**Protein symbol**	Spectral count - Proteomic			p-value - Proteomic		Fold change - Proteomic		Log2 Fold change - Proteomic	
		shCTL	shITGA5	shITGB3	shITGA5	shITGB3	shITGA5	shITGB3	shITGA5	shITGB3
Casein kinase 2 alpha 2	CSNK2A2	5,67	3,00	3,33	**0,031**	**0,054**	0,60	0,60	**-0,74**	**-0,74**
Heat shock protein family A (Hsp70) member 4 like	HSPA4L	21,33	26,33	25,67	**0,016**	**0,048**	1,40	1,30	**0,49**	**0,38**
Importin 8	IPO8	5,00	2,67	3,00	**0,024**	**0,048**	0,60	0,60	**-0,74**	**-0,74**
Small nuclear ribonucleoprotein polypeptides B and B1	SNRPB	19,00	13,33	13,33	**0,066**	**0,035**	0,80	0,70	**-0,32**	**-0,51**
Serine and arginine rich splicing factor 6	SRSF6	5,33	8,33	6,67	**0,063**	**0,018**	1,70	1,30	**0,77**	**0,38**
Transportin 2	TNPO2	9,00	5,33	6,00	**0,087**	**0,067**	0,70	0,70	**-0,51**	**-0,51**

Additional table 2: Selected gene descriptions

Gene name	Gene symbol	Gene description and role in cancer
**Ataxin 2**	**ATXN2**	Ataxin 2 (ATXN2 – GO category "RNA localization" – RNAseq data) was found upregulated in late migrating cells (FC= 7,14E+14) and in migrating cells (FC= 6,39E+14). ATXN2 is involved in negative regulation of EGFR endocytosis and as a RNA-binding protein by regulating their stability. It is implicated in spinocerebellar ataxias type II (SCA2) and amyotrophic lateral sclerosis (ALS)[41,42]. ATXN2 was recently shown to regulate ITGB3 expression, inducing the accumulation of this integrin in cytoplasm of platelets from mice deficient for Atxn2, hence provoking the reduction of their αIIbβ3 integrin-mediated aggregation capacity[43]. ATXN2 is also considered as an unfavorable prognostic marker in liver cancer[19].
**Casein kinase 2 alpha 2**	**CSNK2A2**	Casein kinase 2 alpha 2 (CSNK2A2 – GO categories "Protein folding", "Protein targeting" and "Etablishment of protein localization to organelle" – RNAseq and proteomic data) was found down regulated in late migrating cells (FC= -0.93) and in both shITGA5 (FC= -0.74) and shITGB3 cells (FC= -0.74). CSNK2A2 encodes for the catalytic subunit of the serine/threonine protein casein kinase 2 (CK2). CK2 is involved in multiple cellular processes such as cell cycle, apoptosis and transcription. The later is explained by the direct phosphorylation of RNA polymerases I, II, III and IV but also of numerous transcription factors (STAT1/3, JUN, FOS, MYC, BRCA1…)[44,45,46]. CK2 alpha subunits were described to favor cell adhesion through Akt-GSK3β axis in GN11 neuronal cell line. CSNK2A2 is also considered as an unfavorable prognostic marker in head and neck and liver cancers[19].
**Heat shock protein family A (Hsp70) member 8**	**HSPA8**	Heat shock protein family A (Hsp70) member 8 (HSPA8 – GO categories "Protein folding" and "RNA splicing" – RNAseq data) was found downregulated in migrating cells (FC= -8,01E+14). HSPA8 is a chaperone protein involved into multiple cellular processes, notably in protein folding and degradation regulation. It carries an ATPase activity and is involved in

		regulating autophagy under hypoxia[47,48,49,50]. Hsp70-dependent activation of FAK-Src signaling was shown to be involved in modulating migration and invasion capacity in glioma cell lines[51]. HSPA8 overexpression was highlighted in metastatic melanoma when compared to primary melanomas[52]. Recently, data mining connected HSPA8 to a poor prognosis in TNBC and HPA8 expression was also found dysregulated in ductal carcinoma in situ and invasive breast cancer[53,54]. HSPA8 is considered as an unfavorable prognostic maker in colorectal and renal cancers and as favorable prognostic marker in liver cancer[19].
**RNA binding fox-1 homolog 1**	**RBFOX1**	RNA binding fox-1 homolog 1 (RBFOX1 or ataxin-2-binding protein – GO categories “RNA localization” and “RNA splicing – RNAseq data) was found upregulated in migrating cells (FC= 6,62E+13) and between late and migrating cells (FC= -0.61). RBFOX1 is a highly conserved protein implied in RNA and microRNA splicing, binding to UGCAUG RNA element, and involved in neuronal development, autism spectrum disorder and skeletal muscle development[55,56,57,58,59,60]. RBFOX1 was found to interact with ATXN2 in human neuronal cells with a potential role in RNA transport[60]. RBFOX1 expression is sufficient to promote partial mesenchymal phenotype in breast cancer cells, notably by favoring filamin-B (FLNB) skipping exon 30 variant[61]. However, RBFOX1 has not been considered as a biomarker in any cancer until now[19].
**SMG7 nonsense-mediated mRNA decay factor**	**SMG7**	SMG7 nonsense-mediated mRNA decay factor (SMG7 – GO category “RNA localization” – RNAseq data) was found upregulated in migrating cells (7,42E+14). SMG7 is an essential element of the complex of detection and rapid degradation of transcripts with premature stop codons[62]. SMG7 also had a function in maintenance of telomere integrity[63]. SMG7 was related to regulation of p53 function, notably during p53-mediated response to genotoxic stress by regulating p53 stability in cancer cells[64]. SMG7 is expressed in all cancers and is not considered as a biomarker[19].

**Heat shock protein family A (Hsp70) member 4 like**	**HSPA4L**	Heat shock protein family A (Hsp70) member 4 like (HSPA4L – GO category "Protein folding" – proteomic data) was found upregulated in both shITAG5 (FC= 0,49) and shITGB3 (FC= 0,38) invalidated cells. HSPA4L is a chaperone involved in protection against proteins aggregates. In nasopharyngeal carcinoma, silencing of HSPA4L mediated by miR-497 suppressed cell proliferation and migration and induced apoptosis[65]. HSPA4L is considered as an unfavorable prognostic marker in urothelial cancer and as a favorable prognostic marker in renal cancer[19].
**Importin 8**	**IPO8**	Importin 8 (IPO8 – GO category "establishment of protein localization to organelle" – proteomic data) was found downregulated in both shITAG5 (FC= -0.74) and shITGB3 (FC= -0.74) invalidated cells. IPO8 is a RanGTP binding protein that can act as an autonomous nuclear transport receptor or as part of the importin-α/β complex involved in nuclear import of proteins containing a nuclear localization signal[66]. In TNBC and estrogen receptor-negative breast cancer patients, IPO8 permits the miR551b-3p nucleus translocation, that activates STAT3 and Oncostatin M (OSM), favoring tumor growth and cell migration and invasion[67]. IPO8 is considered as an unfavorable prognostic marker in liver cancer and as favorable prognostic marker in renal cancer[19].
**Small nuclear ribonucleoprotein polypeptides B and B1**	**SNRPB**	Small nuclear ribonucleoprotein polypeptides B and B1 (SNRPB – GO categories " RNA splicing" and "establishment of protein localization to organelle" – proteomic data) was found downregulated in both shITAG5 (FC= -0.32) and shITGB3 (FC= -0.51) invalidated cells. SNRPB is a component of small nuclear ribonucleoprotein spliceosomal complex and plays a role in pre-mRNA splicing. Single nucleotide polymorphism (SNP) of SNRPB (rs6138178) was commonly found in patients with mutations in the BRCA1 gene and its detection can be part in the improvement of breast cancer risk assessement[68]. A recent study showed that RAB26 splicing SNRPB-mediated was necessary to maintain cell growth and migration capacity in non-small cell lung carcinoma (NSCLC)[69]. SNRPB is considered as an unfavorable prognostic marker in liver cancer[19].

**Transportin 2**	**TNPO2**	Transportin 2 (TNPO2 – GO category “establishment of protein localization to organelle” – proteomic data) was found downregulated in both shITAG5 (FC= -0.51) and shITGB3 (FC= -0.51) invalidated cells. TNPO2 problably plays a role in nuclear protein transport, in Ran-GTPase-dependent manner, and is also implicated in apoptosis regulation[70,71]. TNPO2 was also described to promote gastric cancer cell proliferation and apoptosis inhibition through DYNC1I1 dosnwstream[72]. TNPO2 is considered as an unfavorable prognostic marker in endometrial cancer and as favorable prognostic marker in renal cancer[19].

Additional table 3: Sequences of the primers used for quantitative PCR

Gene	Primer	Sequence	% GC	Tm °C	Amplicon size (bp)
ITGB3	ITGB3-F-h	CCGTGACGAGATTGAGTCA	53	56.91	132
	ITGB3-R-h	AGGATGGACTTTCCACTAGAA	43	55.69	
ITGA5	ITGA5-F-h	TGCAGTGTGAGGCTGTGTACA	52	61.91	88
	ITGA5-R-h	GTGGCCACCTGACGCTCT	67	62.01	
IPO8	IPO8-F-h	CGAATTCAGTTGCCTCACAA	45	59.84	155
	IPO8-R-h	ACTGCAGGAGGTCGATTTTG	50	60.25	
SNRPB	SNRPB-F-h	GCACCTTCAAGGCTTTTGAC	50	59.86	166
	SNRPB-R-h	CCCTCTACTGTCATTGAGACCA	50	59.21	
SRSF6	SRSF6-F-h	GAGCTGAACGGCAAGGAG	61	59.66	161
	SRSF6-R-h	CGAACAGGTGGTCCGTATTT	50	59.85	
CSNK2A2	CSNK2A2-F-h	TGAGGCCCTAGATCTTCTGG	55	57.91	175
	CSNK2A2-R-h	TTCCAGTCTTCATCGTGCTG	50	57.92	
HSPA4L	HSPA4L-F-h	CCACAAGAAGGAACCATTTGA	43	56.34	163
	HSPA4L-R-h	TCCATGGATGTTAACACGAACT	41	57.72	
TNPO2	TNPO2-F-h	CTCAACCAGCCGGAATACAT	50	57.67	165
	TNPO2-R-h	CTGGTAGACGGGCTCACAGT	60	61.25	
HSPA8	HSPA8-F-h	CTCGTATCCCCAAGATTCAGA	48	56.62	169
	HSPA8-R-h	GTGACATCCAAGAGCAGCAA	50	58.47	
RBFOX1	RBFOX1-F-h	TTCGAAAATAGTGCCGATGC	45	56.89	170
	RBFOX1-R-h	TAGACTGCACCCACAACTGG	55	59.6	
SMG7	SMG7-F-h	TAGTGCGACACCACTTCCAG	55	59.68	152
	SMG7-R-h	AGATCAAGCGTTGCTGTCGT	50	60.32	
ATXN2	ATXN2-F-h	AAAGACGCAGCTGAGCAAGT	50	60.53	163
	ATXN2-R-h	CTGGAGTTGGCTGTTGATGA	50	57.81	
α-tubulin	Tub-178F	CCCGAGGGCACTACACCAT	63	60.00	108
	Tub-323R	CAGGGAGGTGAACCCAGAAC	60	60.00	
ATXN2 var 3	ATXN2-3-F-h	ATGATGGCACCACCAACACA	50	60.18	105
	ATXN2-3-R-h	GGAGCCCGTGGAAACATACA	55	60.04	
ATXN2 var 2	ATXN2-2-F-h	TGTGGGCTAGAGATGCGACT	55	60.68	129
	ATXN2-2-R-h	GGGTGGCTGTGTCGTCATTA	55	60.04	
ATXN2 var 5	ATXN2-5-F-h	CAACTTCACCTCGGCCTCAA	55	60.25	214
	ATXN2-5-R-h	GGCATATTTGGTACTTTACCTGC	43.48	57.82	
ATXN2 tot	ATXN2-tot-F-h	TGCACCCACATACTCCACAC	55	59.96	166
	ATXN2-tot-R-h	TGGTAAATGGCTGACTGCTG	50	58.19	
MAP2-Total	MAP2-h-tot-F-1	CCTGGGTCTACTGCCATCAC	60	59.82	227
	MAP2-h-tot-R-1	TTCAGGTCTGGCAGTGGTTG	55	60.18	
MAP2-Short	MAP2-h-short-F	TCCGGGGCTAGAAAGAGTGA	55	59.96	73
	MAP2-h-short-R	CTTGCAGACACCTCCTCTCG	60	60.11	
MAP2-Medium	MAP2-h-med-F	ACCCTTTGAGAACACGACACA	47.62	59.79	170
	MAP2-h-med-R	AGGTGGATGTGAGTGTGCAG	55	59.96	

Additional table 4: Antibodies used for western-blotting (WB) and immunofluorescence labeling (IF)

Antibody / Probe	Reference	Species	Dilution WB	Dilution IF
Anti-integrin alpha 5	Ab150361 (Abcam)	Rabbit	1/1000	1/100
Anti-integrin beta 3	sc-365679 (Santa Cruz)	Mouse	1/1000	1/50
Anti-SRSF6	PA5-41810 (Thermo Fisher)	Rabbit	1/1000	
Anti-LAMP1	H4A3.c (Hybridoma Bank)	Mouse	-	1/100
Anti-ALIX	2171 (Cell Signalling Technology)	Rabbit	-	1/100
Anti-alpha-tubulin	T5168 (Sigma-Aldrich)	Mouse	1/10000	-
Phalloidin (F-Actin) Alexa 568 nm	A-1280 (Thermo Fisher)	-	-	1/100
Anti-mouse Alexa 568 nm	A-11004 (Thermo Fisher)	-	1/10000	1/1000
Anti-rabbit Alexa 488 nm	A-11008 (Thermo Fisher)	-	1/10000	1/1000
Hoechst	H-21491 (Thermo Fisher)	-	-	1/2000

References

41. Nonis, D., Schmidt, M.H.H., van de Loo, S., Eich, F., Dikic, I., Nowock, J., and Auburger, G. (2008). Ataxin-2 associates with the endocytosis complex and affects EGF receptor trafficking. Cell. Signal. *20*, 1725–1739. 10.1016/j.cellsig.2008.05.018.

42. Ostrowski, L.A., Hall, A.C., and Mekhail, K. (2017). Ataxin-2: From RNA control to human health and disease. Genes (Basel). *8*, 2–21. 10.3390/genes8060157.

43. Hansen, M., Zeddies, S., Meinders, M., Di Summa, F., Rollmann, E., van Alphen, F.P.J., Hoogendijk, A.J., Moore, K.S., Halbach, M., Gutiérrez, L., et al. (2020). The RNA-binding protein ATXN2 is expressed during megakaryopoiesis and may control timing of gene expression. Int. J. Mol. Sci. *21*, 1–20. 10.3390/ijms21030967.

44. Rozovski, U., Harris, D.M., Li, P., Liu, Z., Jain, P., Veletic, I., Ferrajoli, A., Burger, J., O'brien, S., Bose, P., et al. (2017). Constitutive phosphorylation of STAT3 by the CK2-BLNK-CD5 complex. Mol. Cancer Res. *15*, 610–618. 10.1158/1541-7786.MCR-16-0291.

45. Vilk, G., Weber, J.E., Turowec, J.P., Duncan, J.S., Wu, C., Derksen, D.R., Zien, P., Sarno, S., Donella-Deana, A., Lajoie, G., et al. (2008). Protein kinase CK2 catalyzes tyrosine phosphorylation in mammalian cells. Cell. Signal. *20*, 1942–1951. 10.1016/j.cellsig.2008.07.002.

46. O'Brien, K.A., Lemke, S.J., Cocke, K.S., Rao, R.N., and Beckmann, R.P. (1999). Casein kinase 2 binds to and phosphorylates BRCA1. Biochem. Biophys. Res. Commun. *260*, 658–664. 10.1006/bbrc.1999.0892.

47. Finka, A., Sharma, S.K., and Goloubinoff, P. (2015). Multi-layered molecular mechanisms of polypeptide holding, unfolding and disaggregation by HSP70/HSP110 chaperones. Front. Mol. Biosci. *2*, 29. 10.3389/fmolb.2015.00029.

48. O'Donnell, J.P., Marsh, H.M., Sondermann, H., and Sevier, C.S. (2018). Disrupted Hydrogen-Bond Network and Impaired ATPase Activity in an Hsc70 Cysteine Mutant. Biochemistry *57*, 1073–1086. 10.1021/acs.biochem.7b01005.

49. Hubbi, M.E., Hu, H., Kshitiz, Ahmed, I., Levchenko, A., and Semenza, G.L. (2013). Chaperone-mediated autophagy targets hypoxia-inducible factor-1β(HIF- 1β) for lysosomal degradation. J. Biol. Chem. *288*, 10703–10714. 10.1074/jbc.M112.414771.

50. Robert, G., Jacquel, A., and Auberger, P. (2019). Chaperone-Mediated Autophagy and Its Emerging Role in Hematological Malignancies. Cells *8*, 1260. 10.3390/cells8101260.

51. Sun, G., Cao, Y., Xu, Y., Huai, D., chen, P., Guo, J., Li, M., and Dai, Y. (2019). Overexpression of Hsc70 promotes proliferation, migration, and invasion of human glioma cells. J. Cell.

Biochem. *120*, 10707–10714. 10.1002/jcb.28362.

52. Carta, F., Demuro, P.R., Zanini, C., Santona, A., Castiglia, D., D'Atri, S., Ascierto, P.A., Napolitano, M., Cossu, A., Tadolini, B., et al. (2005). Analysis of candidate genes through a proteomics-based approach in primary cell lines from malignant melanomas and their metastases. Melanoma Res. *15*, 235–244. 10.1097/00008390-200508000-00002.

53. Chen, J., Liu, C., Cen, J., Liang, T., Xue, J., Zeng, H., Zhang, Z., Xu, G., Yu, C., Lu, Z., et al. (2020). KEGG-expressed genes and pathways in triple negative breast cancer. Medicine (Baltimore). *99*, e19986. 10.1097/MD.0000000000019986.

54. Hou, L., Chen, M., Wang, M., Cui, X., Gao, Y., Xing, T., Li, J., Deng, S., Hu, J., Yang, H., et al. (2016). Systematic analyses of key genes and pathways in the development of invasive breast cancer. Gene *593*, 1–12. 10.1016/j.gene.2016.08.007.

55. Conboy, J.G. (2017). Developmental regulation of RNA processing by Rbfox proteins. Wiley Interdiscip. Rev. RNA *8*. 10.1002/wrna.1398.

56. Sun, S., Zhang, Z., Fregoso, O., and Krainer, A.R. (2012). Mechanisms of activation and repression by the alternative splicing factors RBFOX1/2. RNA *18*, 274–283. 10.1261/rna.030486.111.

57. Fogel, B.L., Wexler, E., Wahnich, A., Friedrich, T., Vijayendran, C., Gao, F., Parikshak, N., Konopka, G., and Geschwind, D.H. (2012). RBFOX1 regulates both splicing and transcriptional networks in human neuronal development. Hum. Mol. Genet. *21*, 4171–4186. 10.1093/hmg/dds240.

58. Bill, B.R., Lowe, J.K., DyBuncio, C.T., and Fogel, B.L. (2013). Orchestration of neurodevelopmental programs by RBFOX1: Implications for autism spectrum disorder. In International Review of Neurobiology (Academic Press Inc.), pp. 251–267. 10.1016/B978-0-12-418700-9.00008-3.

59. Hamada, N., Ito, H., Nishijo, T., Iwamoto, I., Morishita, R., Tabata, H., Momiyama, T., and Nagata, K.I. (2016). Essential role of the nuclear isoform of RBFOX1, a candidate gene for autism spectrum disorders, in the brain development. Sci. Rep. *6*. 10.1038/srep30805.

60. Shibata, H., Huynh, D.P., and Pulst, S.M. (2000). A novel protein with RNA-binding motifs interacts with ataxin-2. Hum. Mol. Genet. *9*, 1303–1313. 10.1093/hmg/9.9.1303.

61. Li, J., Choi, P.S., Chaffer, C.L., Labella, K., Hwang, J.H., Giacomelli, A.O., Kim, J.W., Ilic, N., Doench, J.G., Ly, S.H., et al. (2018). An alternative splicing switch in FLNB promotes the mesenchymal cell state in human breast cancer. Elife *7*. 10.7554/eLife.37184.

62. Unterholzner, L., and Izaurralde, E. (2004). SMG7 acts as a molecular link between mRNA surveillance and mRNA decay. Mol. Cell *16*, 587–596. 10.1016/j.molcel.2004.10.013.

63. Azzalin, C.M., Reichenbach, P., Khoriauli, L., Giulotto, E., and Lingner, J. (2007). Telomeric

repeat-containing RNA and RNA surveillance factors at mammalian chromosome ends. Science (80-.). *318*, 798–801. 10.1126/science.1147182.

64. Luo, H., Cowen, L., Yu, G., Jiang, W., and Tang, Y. (2016). SMG7 is a critical regulator of p53 stability and function in DNA damage stress response. Cell Discov. *2*, 15042. 10.1038/celldisc.2015.42.

65. Wang, S., Mo, Y., Midorikawa, K., Zhang, Z., Huang, G., Ma, N., Zhao, W., Hiraku, Y., Oikawa, S., and Murata, M. (2015). The potent tumor suppressor miR-497 inhibits cancer phenotypes in nasopharyngeal carcinoma by targeting ANLN and HSPA4L. Oncotarget *6*, 35893–35907. 10.18632/oncotarget.5651.

66. Görlich, D., Dabrowski, M., Bischoff, F.R., Kutay, U., Bork, P., Hartmann, E., Prehn, S., and Izaurralde, E. (1997). A novel class of RanGTP binding proteins. J. Cell Biol. *138*, 65–80. 10.1083/jcb.138.1.65.

67. Parashar, D., Geethadevi, A., Aure, M.R., Mishra, J., George, J., Chen, C., Mishra, M.K., Tahiri, A., Zhao, W., Nair, B., et al. (2019). miRNA551b-3p Activates an Oncostatin Signaling Module for the Progression of Triple-Negative Breast Cancer. Cell Rep. *29*, 4389-4406.e10. 10.1016/j.celrep.2019.11.085.

68. Wang, X., Pankratz, V.S., Fredericksen, Z., Tarrell, R., Karaus, M., McGuffog, L., Pharaoh, P.D.P., Ponder, B.A.J., Dunning, A.M., Peock, S., et al. (2010). Common variants associated with breast cancer in genome-wide association studies are modifiers of breast cancer risk in BRCA1 and BRCA2 mutation carriers. Hum. Mol. Genet. *19*, 2886–2897. 10.1093/hmg/ddq174.

69. Liu, N., Wu, Z., Chen, A., Wang, Y., Cai, D., Zheng, J., Liu, Y., and Zhang, L. (2019). SNRPB promotes the tumorigenic potential of NSCLC in part by regulating RAB26. Cell Death Dis. *10*, 667. 10.1038/s41419-019-1929-y.

70. Siomi, M.C., Eder, P.S., Kataoka, N., Wan, L., Liu, Q., and Dreyfuss, G. (1997). Transportin-mediated nuclear import of heterogeneous nuclear RNP proteins. J. Cell Biol. *138*, 1181–1192. 10.1083/jcb.138.6.1181.

71. Von Roretz, C., Macri, A.M., and Gallouzi, I.E. (2011). Transportin 2 regulates apoptosis through the RNA-binding protein HuR. J. Biol. Chem. *286*, 25983–25991. 10.1074/jbc.M110.216184.

72. Gong, L., Wen, T., Li, Z., Wang, Y., Wang, J., Che, X., Liu, Y., and Qu, X. (2019). TNPO2 operates downstream of DYNC1I1 and promotes gastric cancer cell proliferation and inhibits apoptosis. Cancer Med. *8*, 7299–7312. 10.1002/cam4.2582.
